# Can the Analysis of Toll-like Receptors (TLR) on NK and NKT-like Cells Improve Gastric Cancer Diagnostics and Treatment?

**DOI:** 10.3390/cancers16223854

**Published:** 2024-11-17

**Authors:** Marek Kos, Krzysztof Bojarski, Paulina Mertowska, Sebastian Mertowski, Piotr Tomaka, Monika Zaborek-Łyczba, Jakub Łyczba, Łukasz Dziki, Ewelina Grywalska

**Affiliations:** 1 Department of Public Health, Medical University of Lublin, 1 Chodźki Street, 20-093 Lublin, Poland; 2 General Surgery Department, Independent Public Health Care Center in Łęczna (SP ZOZ in Leczna), 52 Krasnystawska Street, 21-010 Leczna, Poland; 3 Department of Experimental Immunology, Medical University of Lublin, 4a Chodźki Street, 20-093 Lublin, Poland; sebastian.mertowski@umlub.pl (S.M.); ewelina.grywalska@umlub.pl (E.G.); 4 Department of Anesthesiology and Intensive Care, Independent Public Health Care Center in Łęczna (SP ZOZ in Leczna), 52 Krasnystawska Street, 21-010 Leczna, Poland; 5 Department of General and Oncological Surgery, Faculty of Medicine, Medical University of Lodz, 251 Street, 92-213 Lodz, Poland

**Keywords:** gastric cancer, Toll-like receptors, NK cells, NKT-like cells, RT-qPCR, cancer subtypes, biomarkers, immunotherapy

## Abstract

**Background/Objectives**: The aim of this study was to determine the assessment of the percentage of NK and NKT-like cells expressing Toll-like receptors (TLR-2, TLR-3, TLR-4, and TLR-9) in patients with gastric cancer (GC) compared with healthy volunteers (HV) and to investigate differences according to cancer subtype. We also assessed TLR gene expression by RT-qPCR to assess whether TLRs could be diagnostic and prognostic biomarkers. **Methods**: The study included 86 patients with histologically confirmed gastric cancer and 30 healthy volunteers. Peripheral blood samples were collected from the participants, and TLR expression on NK and NKT-like cells was assessed by flow cytometry and RT-qPCR. The expression of TLR2, TLR3, TLR4, and TLR9 genes was assessed using genetic material derived from NK and NKT-like cells sourced from PBMC. The obtained results were statistically analyzed using Mann–Whitney U and Kruskal–Wallis tests, and the predictive ability of variables was assessed using ROC curve analysis. **Results**: A significantly higher expression of TLR receptors (TLR-2, TLR-3, TLR-4, and TLR-9) was found in patients with gastric cancer compared to healthy volunteers (*p* < 0.05). TLR expression also differed depending on the cancer subtype, and higher expression was observed in more advanced GC subtypes. RT-qPCR analysis showed significantly increased expression of TLR genes in the group of GC patients. ROC curves indicate a high ability of TLRs to differentiate between GC patients and healthy individuals. **Conclusions**: The expression of TLRs on NK and NKT-like cells is clearly increased in patients with gastric cancer, especially in more advanced subtypes of the tumor. The results suggest that TLRs could potentially be used as diagnostic and prognostic biomarkers and represent potential targets for immune therapies in GC. However, further studies are needed to determine the functional role of TLRs in disease progression and the possibility of their use in personalized treatment.

## Simple Summary

In this study, we evaluated the expression of Toll-like receptors (TLRs) on NK and NKT-like cells in patients with gastric cancer (GC) in comparison to its two subtypes: intestinal and diffuse, and in healthy volunteers. We showed that TLR expression increases with disease progression, which may be related to the adaptive immune response to tumor progression. Furthermore, we identified differences in TLR expression depending on the gastric cancer subtype. RT-qPCR analysis revealed significantly higher expression of TLR2, TLR3, TLR4, and TLR9 genes in patients with GC. The results suggest that TLR expression levels may represent a valuable diagnostic and prognostic biomarker and potentially support the personalization of treatment and the development of new immunotherapy strategies in the future.

## 1. Introduction

Despite the development of new therapeutic and diagnostic strategies and even improved patient outcomes, stomach cancer is still a challenge, especially in advanced stages of cancer. In 2018, more than 1 million patients were diagnosed with stomach cancer worldwide, which is 5.7% of all cancer patients. Stomach cancer is aggressive, and 40% of patients have metastatic disease at diagnosis [1]. Gastric cancer occurs in two main subtypes: intestinal type and diffuse type, which differ molecularly, epidemiologically, and clinically. The intestinal type is characterized by an organized cell structure resembling intestinal epithelium. It occurs mainly in older people and develops gradually, passing through stages of inflammation, atrophic mucositis, and intestinal metaplasia [2,3]. The diffuse type, on the other hand, occurs more often in younger people and has a more aggressive course. It is associated with mutations in the CDH1 gene, which leads to a loss of adhesion between cells. Cancer cells of this type are more diffuse and spread throughout the stomach wall, leading to their thickening. For this reason, this type of cancer is more difficult to detect in the early stages and has a worse prognosis [2]. Therefore, research is constantly being conducted to detect this cancer earlier or to develop more effective treatment regimens. For this purpose, numerous studies are being conducted on the influence of the immune system on the development and treatment of stomach cancer. One of the elements of the immune system taking part in the fight against cancer is Natural killer (NK) cells, which, being an element of the innate response, are also able to influence the adaptive response, thus constituting a kind of bridge between these two types of immunity [4]. Due to the influence of these cells not only on other cells through the secretion of cytokines, they can also actively destroy infected or degenerated tumor cells. At the same time, it is indicated that in the case of tumors, the number of NK cells infiltrating into the tumor sites is usually reduced, and the function and activation of NK cells are severely inhibited, which promotes the progression and metastasis of tumors [5]. Reduced NK cell cytolytic activity and cytokine secretion capacity are significantly associated with poor prognosis in various cancers. In turn, increased NK cell activation and high NK cell lysis capacity correlate positively with more prolonged overall survival and a better prognosis for patients [5].

Therefore, our study aims to evaluate the expression of Toll-like receptors (TLR-2, TLR-3, TLR-4, and TLR-9) on NK and NKT-like cells in patients with gastric cancer (GC), compare these results with healthy volunteers (HV), and investigate variants depending on the cancer subtype. Furthermore, we aimed to evaluate TLR gene expression using RT-qPCR to investigate their potential as diagnostic and prognostic biomarkers, with particular attention to the expression levels of these TLRs in patients with intestinal and diffuse gastric cancer subtypes, analyze their correlation with the stages of cancer progression, and compare the results in different patient groups and with the control group.

## 2. Materials and Methods

### 2.1. Research Material and Criteria for Patient Inclusion and Exclusion from the Study

The study included 86 patients with histologically confirmed diagnosis of gastric cancer (GC), regardless of the stage of the disease (I–IV), aged 18 years and older, with a performance status of 0–2 according to the Eastern Cooperative Oncology Group (ECOG) scale. All patients gave written, informed consent to participate in the study after familiarizing themselves with the purpose, scope, and potential risks of the study. Additionally, only peripheral blood in EDTA (10 mL) was collected from them for analysis. Exclusion criteria included the presence of active malignancy other than gastric cancer, except for fully cured basal cell carcinoma, squamous cell carcinoma of the skin, or cervical cancer in situ. Patients who had received chemotherapy, radiotherapy, immunotherapy, or other forms of anticancer treatment within the last 4 weeks before entering the study were also excluded, as were those with active autoimmune disease or chronic inflammatory disease that could affect the results of the study. Patients with severe heart, liver, or kidney failure (e.g., creatinine clearance < 30 mL/min, decompensated cirrhosis, NYHA class III-IV heart failure) were not included in the study, as were those with active, uncontrolled infection, including HIV, HBV, or HCV. Patients who were pregnant, breastfeeding, or unwilling to use effective contraception during the study were also excluded. Additionally, patients who did not provide informed consent to participate in the study were not included.

**Figure 1:**
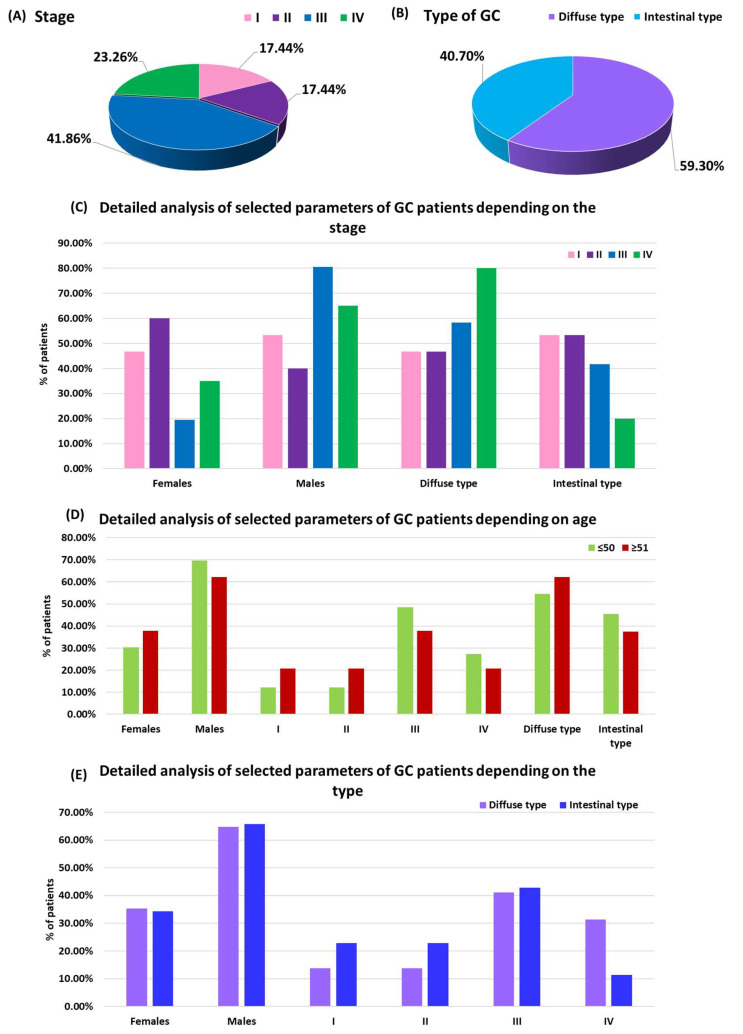
Characteristics of patients recruited to the study: (**A**) Differentiation of patients with GC according to the individual stages; (**B**) Differentiation of patients with GC according to the type of GC; (**C**) Detailed characteristics of selected parameters of patients with GC according to the stage of the disease; (**D**) Detailed characteristics of selected parameters of patients with GC according to age; (**E**) Detailed characteristics of selected parameters of patients with GC according to the type.

The control group included 30 healthy volunteers who met the inclusion criteria and did not meet the exclusion criteria. Inclusion criteria included individuals aged 18 years and older, in good health, with no symptoms of any chronic diseases or inflammatory conditions. Volunteers had to have a performance status of 0 according to the Eastern Cooperative Oncology Group (ECOG) scale. All participants gave written, informed consent to participate in the study after familiarizing themselves with its purpose, scope, and potential risks. Only peripheral blood on EDTA (10 mL) was collected from healthy volunteers for analysis. Exclusion criteria included the presence of any chronic disease, including autoimmune, neoplastic, cardiovascular, liver, and kidney diseases, and active infections, including HIV, HBV, or HCV. Volunteers taking any immunosuppressive, antineoplastic, or anti-inflammatory drugs chronically were also excluded from the study. Individuals who were pregnant, breastfeeding, or not using effective contraception were also excluded. Additionally, participants who did not provide informed consent to participate in the study were not included. Detailed information on patient characteristics is presented in Figure 1 and Supplementary Materials Table S1.

### 2.2. Immunophenotyping

To evaluate the expression of TLR2-9 on NK and NKT-cells, the peripheral blood samples from the participants were incubated with a set of monoclonal anti-human antibodies, including CD45 FITC, CD3 BV510, CD4 BV650, CD8 BV605, CD19 PE-Cy7, CD16 BB700, CD56 BUV661, TLR2 PE, TLR4 BV421, TLR3 PE, and TLR9 BV421 (BD, Franklin Lakes, NJ, USA). BD Horizon™ Brilliant Stain Buffer (BD, Franklin Lakes, NJ, USA) was used to enhance antibody stability, thus improving the signal quality for flow cytometry analysis. Erythrocytes were removed using a lysis buffer (BD, Franklin Lakes, NJ, USA), and cells were washed with BD Pharmingen™ Stain Buffer (BSA) (BD, Franklin Lakes, NJ, USA). For intracellular TLR assessment, the BD Cytofix/Cytoperm™ Fixation/Permeabilization Kit (BD, Franklin Lakes, NJ, USA) was applied. The assays were conducted on a CytoFLEX LX flow cytometer (Beckman Coulter, Indianapolis, IN, USA), and daily quality control was ensured with CytoFLEX Daily QC Fluorosphere reagents to minimize instrument-related variability. The acquisition of events was set to collect 15,000 events from the gate expressing CD45, which was the basis for further analyses (Figure S1). Data were analyzed using Kaluza Analysis software version 2.1 (Beckman Coulter, Indianapolis, IN, USA). 

### 2.3. PBMC Isolation

Peripheral blood was collected from healthy donors/patients into tubes with anticoagulant (EDTA). Blood was diluted 1:1 with physiological saline buffer (PBS) and gently applied onto a Gradisol L density gradient (Aqua-med). Samples were centrifuged at room temperature for 20 min at 400× *g* without inhibition. The peripheral blood mononuclear cell (PBMC) layer was collected from the interface and washed twice with PBS supplemented with 2% bovine serum (FBS) and 2 mM EDTA, centrifugation for 10 min at 300× *g*. PBMC was counted and suspended in MACS buffer (PBS, 2% FBS, 2 mM EDTA).

### 2.4. Separation of NK and NKT-Like Cells

According to the manufacturer’s instructions, NK cells were isolated from PBMC (10^7^ cells/mL) using a commercially available NK Cell Isolation Kit, human (Miltenyi Biotec). The procedure used adverse selection, in which NK cells remained unbound to antibodies. PBMC were incubated with a mixture of antibodies binding markers specific for T cells (CD3), B cells (CD19), monocytes (CD14), granulocytes (CD15), and other nonspecific cells, which were then removed using magnetic beads conjugated with anti-mouse antibodies. The cell suspension was passed through a MACS column in a magnetic field. Purified NK cells were collected in the eluate by washing the column with MACS buffer.

NKT-like cells were isolated from PBMC (10^7^ cells/mL) by a two-step method using a commercially available CD3^+^CD56^+^ NKT Cell Isolation Kit, human (Miltenyi Biotec). In the first step, NK cells and monocytes were magnetically labeled using a cocktail of biotin-conjugated antibodies and Anti-Biotin MicroBeads (Miltenyi Biotec). Labeled cells were removed from the sample by passing them through a MACS^®^ column placed in a magnetic field, which allowed the separation of unbound cells as a fraction enriched in NKT cells.

In the second step, CD3⁺CD56⁺ cells were isolated from the obtained NKT cell-enriched fraction by positive selection using CD56 MicroBeads (Miltenyi Biotec) according to the manufacturer’s protocol. Labeled CD3⁺CD56⁺ cells were retained in the MACS^®^ column, and unbound cells were washed away. Then, the column was removed from the magnetic field, and the isolated NKT-like cells were eluted, obtaining a fraction of high purity and viability, ready for further analysis. After separation, cell purity and viability were assessed by flow cytometry (using antibodies against CD3, CD56, and CD16 (BioLegend). NK cells were identified as CD3^−^CD56^bright^ or CD56^dim^, and NKT-like cells as CD3^+^CD56^+^.

### 2.5. RNA Isolation Using A&A Biotechnology Kit

Total RNA isolation was performed using an RNA isolation kit (A&A Biotechnology, Gdańsk, Poland) according to the manufacturer’s protocol. Cells (or tissues) were lysed by adding an appropriate lysis buffer containing guanidine, which denatures proteins, including RNases, preventing RNA degradation. The mixture was mixed vigorously and incubated for several minutes at room temperature to lyse the cells completely.

After lysis, the suspension was transferred to an RNA-binding column, placed in a collection tube, and centrifuged for 1 min at 12,000× *g*, which allowed nucleic acids to bind to the column membrane. The column was then washed multiple times with special wash buffers, including a buffer containing ethanol, which allowed the removal of contaminants such as proteins, lipids, and DNA residues. After each wash, the column was centrifuged to remove excess fluid effectively. In the last step, the RNA was eluted from the column membrane using an elution buffer warmed to room temperature or RNase-treated water (DEPC-treated water). The column was placed in a clean elution tube and centrifuged for 1 min at 12,000× *g*. The resulting RNA was collected, and its concentration and quality were assessed by spectrophotometry (e.g., NanoDrop) and agarose gel electrophoresis or by microcapillary analyzer (e.g., Agilent Bioanalyzer) to ensure RNA integrity.

The isolated RNA was stored at −80 °C until further use in experiments such as RT-qPCR, RNA sequencing (RNA-seq), or gene expression analysis.

### 2.6. cDNA Synthesis

Isolated RNA was subjected to cDNA synthesis using the iScript™ cDNA Synthesis Kit (Bio-Rad, Hercules, CA, USA) according to the manufacturer’s instructions. The reaction was performed in a volume of 20 µL containing 1 µg RNA, 4 µL 5X iScript Reaction Mix, 1 µL iScript Reverse Transcriptase, and RNase-free water. The reaction was performed in a thermocycler (e.g., Simply Ampli, Applied Biotechnology) according to the following program: RNA denaturation and primerization for 5 min at 25 °C, cDNA synthesis for 20 min at 46 °C, and then reverse transcriptase inactivation for 1 min at 95 °C. The resulting cDNA was stored at −20 °C until further analysis.

### 2.7. RT-qPCR (Real-Time Quantitative PCR)

TLR2, TLR3, TLR4, and TLR9 gene expression analysis was performed by RT-qPCR using SYBR Green dye and SsoAdvanced Universal SYBR^®^ Green Supermix^®^ reagents (Bio-Rad). RT-qPCR reactions were performed in a volume of 20 µL containing: 10 µL SYBR Green Supermix, 1 µM PrimePCR Assay for TLR2 (Unique Assay ID: qHsaCED0036567; Assay Design: exonic; Chromosome Location: 4:154624891-154625028; Amplicon Length: 108; Amplicon Context Sequence: GGATTGTTAGAATTAGAGTTTGATGACTGTACCCTTAATGGAGTTGGTAATTTTAG AGCATCTGATAATGACAGAGTTATAGATCCAGGTAAAGTGGAAACGTTAACAATC CGGAGGCTGCATATTCCAAGGTTTTAC), TLR3 (Unique Assay ID: qHsaCED0046212; Assay Design: exonic; Chromosome Location: 4:187003545-187003664; Amplicon Length: 90; Amplicon Context Sequence: CAGCCTTACAGAGAAGCTATGTTTGGAATTAGCAAACACAAGCATTCGGAATCTG TCTCTGAGTAACAGCCAGCTGTCCACCACCAGCAATACAACTTTCTTGGGACTAA AGTGGACAAA), TLR4 (Unique Assay ID: qHsaCED0037607; Assay Design: exonic; Chromosome Location: 9:120474961-120475080; Amplicon Length: 90; Amplicon Context Sequence: CAAGATTCAAAGTATTTATTGCACAGACTTGCGGGTTCTACATCAAATGCCCCTA CTCAATCTCTCTTTAGACCTGTCCCTGAACCCTATGAACTTTATCCAACCAGGTG CATTTAAAGA), TLR9 (Unique Assay ID: qHsaCED0003672; Assay Design: exonic; Chromosome Location: 3:52259820-52259947; Amplicon Length: 98; Amplicon Context Sequence: CCTACATCCCATGAGGGCCTCACACCTGTCCTCTACCAAGCCCAGGGAGGAGCT AAGGCCCAGAGCTCAGGCAGAGAGCAGGGAGAGATGGGAATTCTGGATAGCAC CAGTAGCGGGTACACCTTGCT) and the GAPDH reference gene (Unique Assay ID: qHsaCED0038674; Assay Design: exonic; Chromosome Location: 12:6647267-6647413; Amplicon Length: 117; Amplicon Context Sequence: GTATGACAACGAATTTGGCTACAGCAACAGGGTGGTGGACCTCATGGCCCACAT GGCCTCCAAGGAGTAAGACCCCTGGACCACCAGCCCCAGCAAGAGCACAAGAG GAAGAGAGAGACCCTCACTGCTGGGGAGTCCCTGCCACAC), 2 µL cDNA (approx. 50 ng) and nuclease-free water to make up the volume. The reaction was performed on a CFX96 Real-Time PCR Detection System (Bio-Rad) with the following thermal profile: polymerase activation for 3 min at 95 °C, followed by 40 cycles consisting of denaturation at 95 °C for 10 s, primer annealing at 60 °C for 30 s, and extension at 72 °C for 30 s. The specificity of the amplification was verified by melting curve analysis in the temperature range from 65 °C to 95 °C, increased by 0.5 °C every 5 s. The results of mRNA expression analysis were normalized to the reference gene (GAPDH) using the 2^−ΔΔCt^ method, and the data were presented as relative expression changes compared to the control sample.

### 2.8. Statistical Analysis

Data were statistically analyzed using Statistica (version 13.5.0.17, TIBCO Software Inc., Palo Alto, CA, USA) and GraphPad Prism (version 5.01 for Windows, GraphPad Software, San Diego, CA, USA). Due to the nonparametric nature of the data, the normality of their distribution was assessed using the Shapiro–Wilk test. In the absence of a normal distribution, appropriate nonparametric tests were used. Comparisons between the two groups were performed using the Mann–Whitney U test, which is a nonparametric test used to compare the medians of two independent groups. The test results were presented as the median and interquartile range (IQR). *p* values < 0.05 were considered statistically significant. Comparisons between multiple groups were performed using the Kruskal–Wallis test, which is used to assess differences between more than two independent groups. In the case of significant results of the Kruskal–Wallis test, additional post-hoc analysis with Bonferroni correction was performed to identify which groups were significantly different from each other. Bonferroni correction reduces type I error during multiple comparisons. Correlation analysis was performed using Spearman’s rank correlation coefficient, which is used to assess the strength and direction of the relationship between two variables with a nonparametric distribution. Correlation results were presented as Spearman’s correlation coefficient (rho) with the corresponding *p* value. *p* values < 0.05 were considered statistically significant. Receiver Operating Characteristic (ROC) curve analysis was performed to assess the ability of selected variables to differentiate between individual study groups. Predictor values for each variable were compared between the two groups, and ROC curves were plotted based on the calculated sensitivity and specificity at different cut-off values. Area Under Curve (AUC) was calculated as a measure of the predictive ability of the variable. AUC values were interpreted as follows: AUC = 0.5 indicated no discrimination, AUC > 0.7 was considered acceptable predictive ability, AUC > 0.8 as good, and AUC > 0.9 as very good discrimination between groups. The AUC value of each ROC curve was compared with the value of 0.5 using a significance test to assess whether the variable was significantly different from random prediction. *p* values < 0.05 were considered statistically significant.

## 3. Results

### 3.1. Evaluation of the Percentage of Occurrence and Expression of TLR-2, -3, -4, and -9 on NK and NKT-like Cells in GC Patients and Healthy Volunteers

In the preliminary analysis, two groups were compared: GC (study group) and HV (control group), focusing on various parameters related to immune cell populations and the percentage of the studied TLRs on NK (CD3−CD56+) and NKT-like (CD3+CD56+) cells. The study aimed to assess the differences in the composition and function of immune cells between these groups. The average age of the subjects in the GC group was 59.52 years, while in the HV group, it was 58.65 years; these differences were not statistically significant (*p* = 0.804). In the analysis of the immune cell population, the percentage of CD45+ cells was similar in both groups (GC: 86.39%, HV: 88.02%, *p* = 0.728), as was the percentage of NK cells (GC: 11.10%, HV: 10.98%, *p* = 0.923). No significant differences were observed between the groups in the CD3-CD56+^dim^ and CD3-CD56+^bright^ NK cell subpopulations (Table 1). A significant difference was observed in the percentage of CD3+CD56+ T cells, which was significantly higher in the GC group (3.73%) compared to the HV group (1.84%, *p* = 0.000), suggesting differences in this T cell subpopulation. CD19+ B cells were also more numerous in the HV group (9.62%) than in the GC group (7.72%, *p* = 0.008) (Table 1). The most significant differences were observed in the expression of TLR-2, TLR-3, TLR-4, and TLR-9 receptors on different cell subpopulations: CD3-CD56+^dim^, CD3-CD56+^bright^, and CD3+CD56+. In each of these subpopulations, TLR expression was significantly higher in the GC group compared to HV (*p* = 0.000 for all parameters analyzed), which may suggest differences in the immune response between the groups. Overall, the results indicate significant differences in the expression of TLR receptors on selected CD3-CD56+ and CD3+CD56+ cell subpopulations between the GC and HV groups, which may have important implications for understanding the mechanisms of the immune response in the studied populations.

In addition, we performed a comparative analysis of the expression levels of the studied TLR-2, -3, -4, and -9 in the genetic material derived from CD3-CD56+ to CD3+CD56+ subpopulations sorted from PBMCs between the GC group and HV to assess differences in the immune response. The results showed a significantly higher expression of all studied TLR receptors in the GC group compared to HV (Table 2). In CD3-CD56+ cells, mean TLR-2 expression was 13.37 ± 10.41 in GC and 1.24 ± 0.84 in HV, TLR-3: 7.65 ± 5.50 in GC and 1.26 ± 0.87 in HV, TLR-4: 12.31 ± 8.99 in GC and 1.27 ± 0.90 in HV, and TLR-9: 12.42 ± 9.53 in GC and 1.26 ± 0.87 in HV, with *p* = 0.000 for all comparisons. Similar patterns were observed in CD3+CD56+ cells, where the mean expression of TLR-2 was 6.78 ± 7.35 in GC and 1.30 ± 0.94 in HV, TLR-3: 7.38 ± 4.93 in GC and 1.38 ± 1.12 in HV, TLR-4: 5.51 ± 4.57 in GC and 1.31 ± 0.98 in HV, and TLR-9: 8.19 ± 5.31 in GC and 1.21 ± 0.80 in HV, with significance at *p* = 0.000 (Table 2). These results indicate a significantly higher expression of TLRs in CD3-CD56+ and CD3+CD56+ cells in the GC group, suggesting differences in immune activation and response in the compared populations. All differences were statistically significant, which underlines their potential importance in the context of immune function.

### 3.2. Influence of Disease Stage on the Prevalence and Expression Level of TLR-2, -3, -4, and -9 CD3-CD56+ and CD3+CD56+ Cells in GC Patients

**Figure 2:**
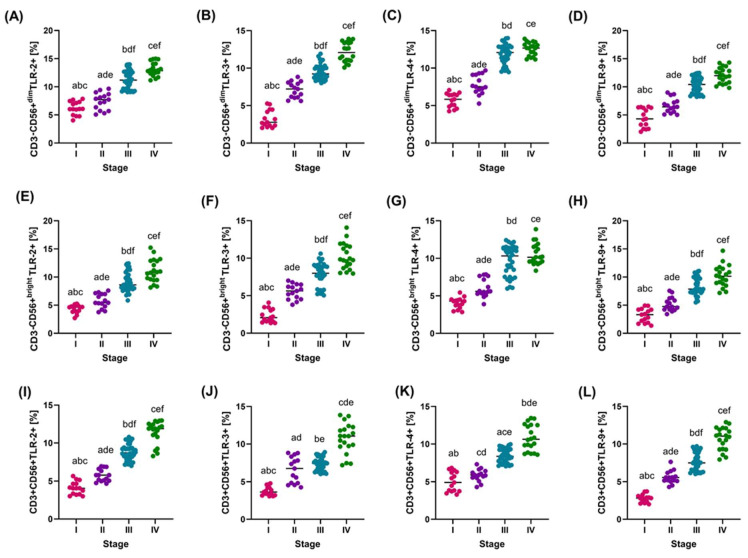
Graphical representation of the percentage of TLR-2, -3, -4, and -9 on individual immune cell subpopulations tested in relation to disease stage (**A**–**D**): Percentage of TLR-2, -3, -4, and -9 on CD56+^dim^; (**E**–**H**): Percentage of TLR-2, -3, -4, and -9 on CD56+^bright^; (**I**–**L**): Percentage of TLR-2, -3, -4, and -9 on CD3+CD56+. For ease of reference, each stage is marked with a different color: pink for stage I; purple for stage II; blue for stage III; and green for stage IV. Statistically significant differences between individual stages are marked on the graph using letters—groups marked with the same letters are statistically significant.

**Figure 3:**
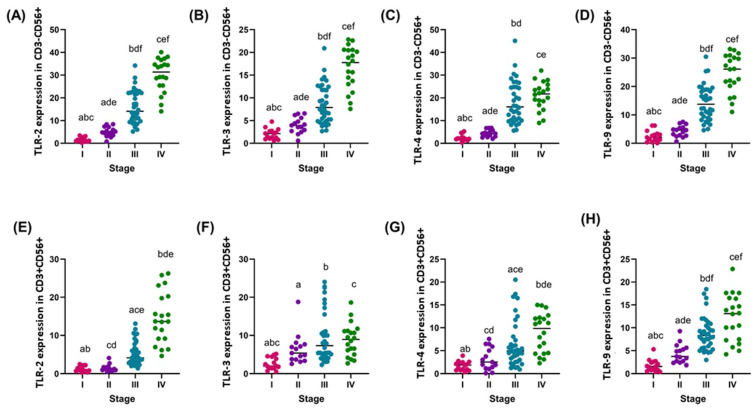
Graphical representation of TLR-2, -3, -4, and -9 expression in genetic material isolated from individual subpopulations of immune cells tested in relation to disease stage. (**A**–**D**) Percentage of TLR-2, -3, -4, and -9 in CD3-CD56+ cells; (**E**–**H**) Percentage of TLR-2, -3, -4, and -9 in CD3+CD56+ cells. For ease of reference, each stage is marked with a different color: pink for stage I; purple for stage II; blue for stage III; and green for stage IV. Statistically significant differences between individual stages are marked on the graph using letters—groups marked with the same letters are statistically significant.

Due to a number of statistically significant changes observed between newly diagnosed GC patients and HV, we decided to take a closer look at the differences occurring within the study group. Particular attention was paid to assessing whether the stage of the disease may significantly affect the changes in the expression profiles of the studied TLR receptors. The study performed a comparative analysis of the obtained test results between the individual stages of GC, the numbers of which in the individual groups were, respectively: 17.44% stage I; 17.44% stage II; 41.86% stage III; and 23.26% stage IV (Figure 1C,E). The obtained data are presented in Supplementary Materials Table S2 and in Figure 2 and Figure 3.

The analysis revealed significant differences in the immune profiles of GC patients depending on the stage of the disease. The age of the patients did not differ significantly between the stages (*p* > 0.05). In the case of CD45+ cells, the percentage was highest in stage II (90.00%) compared to the other stages, and a significant difference was observed only between stages I and II (*p* = 0.050). The percentage of CD3-CD56+ cells showed a clear decrease with disease progression, with the highest value in stage I (15.45%) and the lowest in stage IV (7.84%), and all comparisons between stage I and the other stages were statistically significant (*p* < 0.05). A similar trend was observed in the percentage of CD56+^dim^ cells, which decreased with disease progression, with significant differences between stage I and more advanced stages (*p* < 0.05). The percentage of CD56+^bright^ cells remained relatively stable but showed a significant difference only between stages I and IV (*p* = 0.012) (Supplementary Materials Table S2). CD3+CD56+ cells showed the highest percentage in stage I (6.74%), and their number decreased significantly in stages II, III, and IV, which may suggest reduced activation of these cells in more advanced stages of the disease (*p* < 0.001) (Supplementary Materials Table S2).

Analysis of the percentage of the studied TLRs on different immune cell subpopulations showed a clear increase in their expression with disease progression. TLR-2, TLR-3, TLR-4, and TLR-9 values on CD56+^dim^ and CD56+^bright^ cells increased significantly from stage I to IV (*p* < 0.001), suggesting increasing activation of these receptors in more advanced stages (Figure 2A–H). A similar pattern was observed in TLR expression on CD3+CD56+ cells, where the expression of TLR-2, TLR-3, TLR-4, and TLR-9 increased with disease progression, indicating progressive activation of the immune response (Figure 2I–L).

Analysis of the expression of TLR-2, -3, -4, and -9 receptors on CD3-CD56+ and CD3+CD56+ cells in different disease stages revealed clear differences in expression levels depending on disease stage, which may indicate enhanced immune activation in more advanced stages of GC. TLR-2 expression on CD3-CD56+ cells showed a significant increase, with the median value increasing from 1.29 in stage I to 4.81 in stage II, 12.80 in stage III, and then 27.50 in stage IV, which meant an approximately 3.7-fold increase between stages I and II, 2.7-fold between II and III, and 2.1-fold increase between stages III and IV, respectively (Figure 3A). TLR-3 expression on CD3-CD56+ cells increased from a median of 2.15 in stage I to 3.96 in stage II, 6.69 in stage III, and 15.00 in stage IV, corresponding to a 1.8-fold increase between stages I and II, a 1.7-fold increase between stages II and III, and a 2.2-fold increase between stages III and IV (Figure 3B). TLR-4 expression on CD3-CD56+ cells showed a median increase from 2.11 in stage I to 4.33 in stage II, 13.09 in stage III, and 18.77 in stage IV, corresponding to increases of 2.1-fold between stages I and II, 3.0-fold between II and III, and 1.4-fold between stages III and IV, respectively (Figure 3C). For TLR-9 on CD3-CD56+ cells, median expression increased from 2.07 in stage I to 4.64 in stage II, 12.09 in stage III, and 22.63 in stage IV, corresponding to a 2.2-fold increase between stages I and II, a 2.6-fold increase between II and III, and a 1.9-fold increase between stages III and IV (Figure 3D).

In CD3+CD56+ cells, TLR-2 expression showed stability between stage I (median 1.38) and II (1.21), after which it increased to 4.12 in stage III and 16.21 in stage IV, which represented a significant increase of 3.4-fold between stage II and III and 3.9-fold between stage III and IV (Figure 3E). TLR-3 expression in CD3+CD56+ cells increased gradually from a median of 2.85 in stage I to 5.35 in stage II, 7.55 in stage III, and then 8.95 in stage IV, which represented a 1.9-fold increase between stage I and II, 1.4-fold between stage II and III, and a slight increase between stage III and IV, respectively (Figure 3F). TLR-4 expression increased from a median of 2.10 in stage I to 2.51 in stage II, 4.71 in stage III, and 9.85 in stage IV, which represented a 1.2-fold increase between stages I and II, 1.9-fold between II and III, and 2.1-fold increase between stages III and IV (Figure 3G). TLR-9 expression in CD3+CD56+ cells showed a significant increase from a median of 1.54 in stage I to 4.84 in stage II, 8.27 in stage III, and 14.56 in stage IV, which represented a 3.1-fold increase between stages I and II, a 1.7-fold increase between II and III, and a 1.8-fold increase between stages III and IV (Figure 3H).

### 3.3. Comparative Analysis of the Percentage of Occurrence and Level of Expression of TLR-2, -3, -4, and -9 on CD3-CD56+ and CD3+CD56+ Cells in the Context of Diffuse and Intestinal Type of GC Patients in Relation to HV

**Figure 4:**
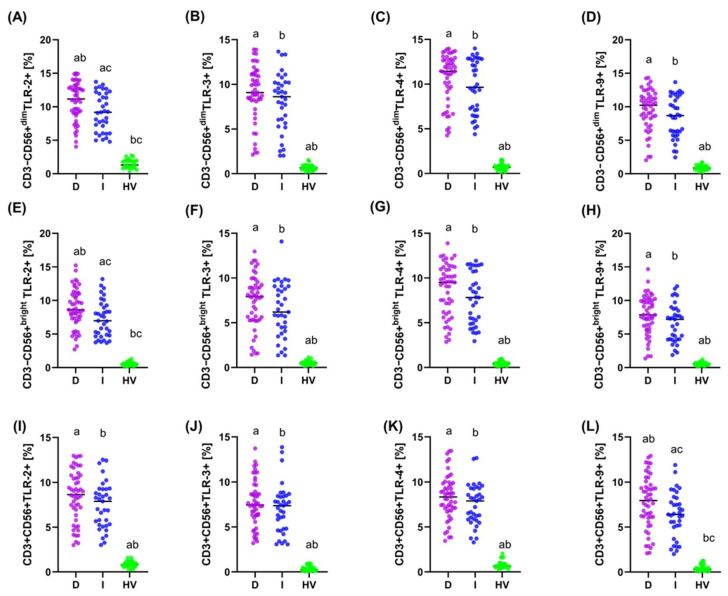
Graphical representation of the percentage of TLR-2, -3, -4, and -9 on individual subpopulations of tested immune cells between GC (diffuse and intestinal type) and HV patients. (**A**–**D**) Percentage of TLR-2, -3, -4, and -9 on CD3-CD56+^dim^; (**E**–**H**) Percentage of TLR-2, -3, -4, and -9 on CD3-CD56+^bright^; (**I**–**L**) Percentage of TLR-2, -3, -4, and -9 on CD3+CD56+. For ease of reference, each group has been marked with a different color: purple diffuse type; blue intestinal type; and green HV. Statistically significant differences between individual stages are marked on the graph using letters—groups marked with the same letters are statistically significant. Abbreviations: D—diffuse type; I—intestinal type; HV—healthy volunteers.

**Figure 5:**
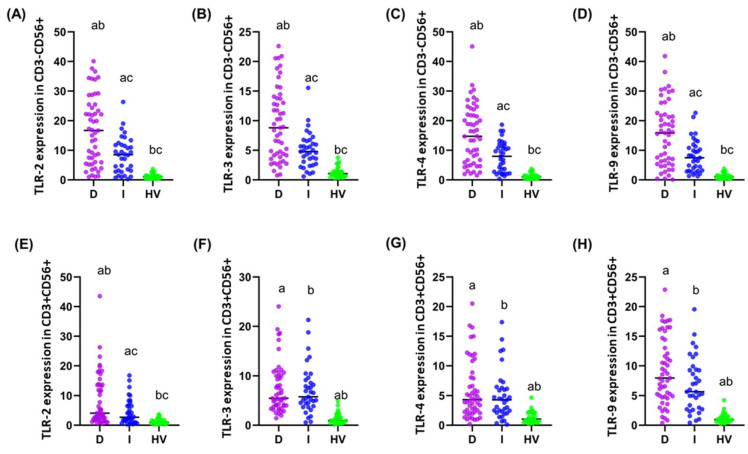
Graphical representation of TLR-2, -3, -4, and -9 expression in genetic material isolated from individual subpopulations of tested immune cells between GC (diffuse and intestinal type) and HV patients (**A**–**D**) Percentage of TLR-2, -3, -4, and -9 on CD3-CD56+^dim^; (**E**–**H**) Percentage of TLR-2, -3, -4, and -9 on CD3-CD56+^bright^; (**I**–**L**) Percentage of TLR-2, -3, -4, and -9 on CD3+CD56+. For convenience, each group was marked with a different color: purple diffuse type; blue intestinal type; and green HV. Statistically significant differences between individual stages are marked on the graph using letters—groups marked with the same letters are statistically significant. Abbreviations: D—diffuse type; I—intestinal type; HV—healthy volunteers.

The comparative analysis of the percentage of occurrence and expression level of the studied TLRs on CD3-CD56+ and CD3+CD56+cells in patients with diffuse and intestinal GC compared to HV was conducted in the next step to assess differences in immune activation between individual histopathological types of cancer, which, as indicated by the literature, may be of significant importance for understanding the mechanisms of the immune response and disease progression. Of the patients recruited to the study, 59.30% were classified as diffuse type, while 40.70% as intestinal type (Figure 1D,G). Detailed data obtained in this analysis are presented in Supplementary Materials Table S3 and presented in Figure 4 and Figure 5. 

In the case of CD3-CD56+ cells, patients with the diffuse type had a significantly lower percentage of these cells (median 9.71%) compared to patients with the intestinal type (median 12.07%, *p* = 0.022) (Supplementary Materials Table S3). Similarly, the percentage of CD3-CD56+^dim^ cells was significantly lower in patients with the diffuse type (median 7.87%) compared to the intestinal type (median 10.59%, *p* = 0.024). In the remaining analyzed parameters, such as the percentages of CD45+, CD3+, CD3+CD56+, CD19 B lymphocytes, CD3+CD4+, CD3+CD8+, and the CD3+CD4+/CD3+CD8+ ratio, no significant differences were observed between patients with the diffuse and intestinal type (Supplementary Materials Table S3). Importantly, the percentage of the studied TLR-2, -3, -4, and -9 receptors was higher in both diffuse and intestinal patients compared to HV (Figure 4). Additionally, statistically significant differences between patients with diffuse to intestinal-type were noted for CD3-CD56+^dim^TLR-2+, CD3-CD56+^bright^TLR-2+, and CD3+CD56+TLR-9+ (Figure 4A,E,L).

The analysis of the expression of the studied TLR-2, -3, -4, and -9 in the genetic material of sorted CD3-CD56+ and CD3+CD56+cells from patients with diffuse and intestinal GC and HV showed significant differences in the expression level. In the case of TLR-2 on CD3-CD56+ cells, the median expression was highest in patients with the diffuse type, which was about twice as high as in the intestinal type and more than 15-fold higher than in HV. The difference between the intestinal type and HV was about 8-fold (Figure 5A). TLR-3 expression on CD3-CD56+ cells showed a similar trend, with a median of 8.80 in the diffuse type, which was 1.8-fold higher than in the intestinal type and about 8.3-fold higher than in HV. The difference between the intestinal type and HV was about 4.5-fold (Figure 5B). For TLR-4 on CD3-CD56+ cells, the median expression in the diffuse type was 14.74, which was approximately 1.8-fold higher than in the intestinal type and 14-fold higher than in HV. Expression in the intestinal type was approximately 7.6-fold higher than in HV (Figure 5C). For TLR-9 on CD3-CD56+ cells, the median expression in the diffuse type was 15.85, which was approximately twice as high as in the intestinal type and approximately 15-fold higher than in HV. Expression in the intestinal type was approximately 7-fold higher than in HV (Figure 5D).

For TLRs on CD3+CD56+ cells, the median expression of TLR-2 was 4.07 in the diffuse type, which was approximately 1.5-fold higher than in the intestinal type and more than 4-fold higher than in HV (Figure 5E). TLR-3 expression on CD3+CD56+ cells was similar between the diffuse and intestinal types, with medians of 5.46 and 5.75, respectively, indicating no significant difference, while both tumor types showed approximately 5-fold higher expression compared to HV (Figure 5F). Similar relationships were observed in the remaining TLRs, where no statistically significant differences were observed between the diffuse and intestinal types (Figure 5G,H).

### 3.4. Influence of Age on the Percentage of Occurrence and Expression Level of TLR-2, -3, -4, and -9 on CD3-CD56+ and CD3+CD56+ Cells in GC Patients in Relation to HV

**Figure 6:**
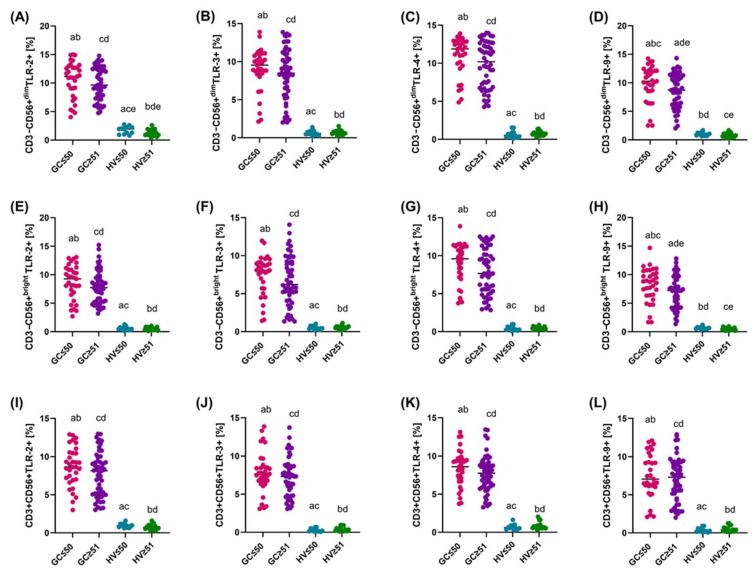
Graphical representation of the percentage of TLR-2, -3, -4, and -9 on individual subpopulations of immune cells tested in relation to patient age (**A**–**D**) Percentage of TLR-2, -3, -4, and -9 on CD3-CD56+^dim^; (**E**–**H**) Percentage of TLR-2, -3, -4, and -9 on CD3-CD56+^bright^; (**I**–**L**) Percentage of TLR-2, -3, -4, and -9 on CD3+CD56+. For convenience, each age group is marked with a different color: pink GC ≤ 50; purple GC ≥ 51; blue HV ≤ 50; and green HV ≥ 51. Statistically significant differences between individual stages are marked on the graph using letters—groups marked with the same letters are statistically significant.

**Figure 7:**
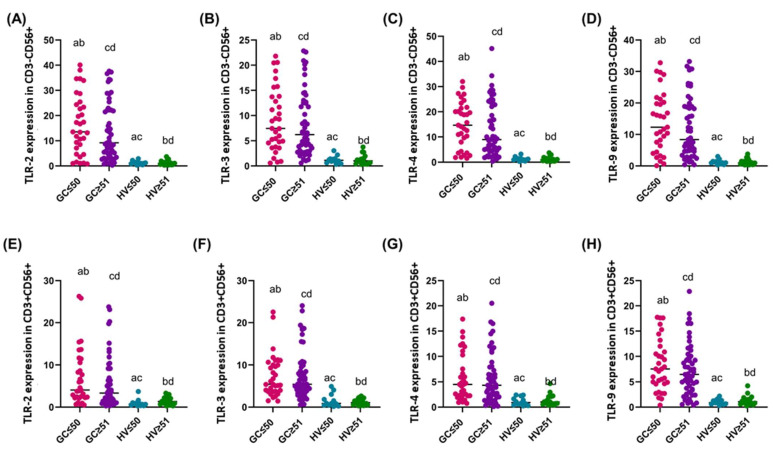
Graphical representation of TLR-2, -3, -4, and -9 expression in genetic material isolated from individual subpopulations of tested immune cells in relation to the age of patients. (**A**–**D**) Percentage of TLR-2, -3, -4, and -9 in CD3-CD56+ cells; (**E**–**H**) Percentage of TLR-2, -3, -4, and -9 in CD3+CD56+ cells. For ease of reference, each age group was marked with a different color: pink GC ≤ 50; purple GC ≥ 51; blue HV ≤ 50; and green HV ≥ 51. Statistically significant differences between individual stages are marked on the graph using letters—groups marked with the same letters are statistically significant.

Age plays an important role in the incidence and progression of GC, therefore, our analyses also considered differences related to the age groups of patients. The analysis was performed in 38.37% of GC patients aged ≤50 years and 61.63% of GC patients aged ≥51 years, as well as in the control group consisting of 40% HV aged ≤50 years and 60% aged ≥51 years (Figure 1D). Similar to the previous analyses, detailed results of the studies are presented in Supplementary Materials Table S4 and graphically in Figure 6 and Figure 7.

The mean age of patients with GC ≤ 50 years was 45.36 ± 3.15 years (median 46.00), while for GC ≥ 51 years, it was 68.34 ± 8.42 years (median 68.00). In the group of HV ≤ 50 years, the mean age was 46.04 ± 3.04 years (median 45.24), and for HV ≥ 51 years, it was 67.06 ± 12.08 years (median 64.61). Significant differences were found between GC ≤ 50 and GC ≥ 51 (*p* = 0.000), indicating a clear age separation between these groups of GC patients. Comparison between GC ≤ 50 and HV ≤ 50 years did not show significant differences (*p* = 0.502), suggesting that the age of patients in the younger groups is comparable between patients and healthy subjects. However, significant differences occurred between GC ≤ 50 and HV ≥ 51 (*p* = 0.000), as well as between GC ≥ 51 and HV ≤ 50 (*p* = 0.000) and HV ≥ 51 and GC ≥ 51, highlighting the influence of age on the results in these groups (Supplementary Materials Table S4).

The results indicate no significant differences in most of the analyzed parameters between younger and older GC patients, except for CD3-CD56+^dim^TLR-9+ and CD3-CD56+^bright^TLR-9+, which were statistically significantly higher in GC patients ≤ 50 (Figure 6D,H). However, a significantly higher percentage of the studied TLRs was demonstrated in both GC groups compared to HV, regardless of age. This suggests that age influences specific immune responses in the context of GC, which may play a key role in disease progression (Figure 6).

The median expression of TLR-2, -3, -4, and -9 was significantly higher in GC patients, both in the ≤50 and ≥51-year-old groups, compared to the HV group, regardless of age (*p* < 0.001 for most comparisons) (Figure 7A–D). A similar trend was observed for the expression of the studied TLRs on CD3+CD56+ cells (Figure 7E–H). However, although significant differences were demonstrated in flow cytometric analyses for CD3-CD56+^dim^TLR-9+ and CD3-CD56+^bright^TLR-9+ between younger and older GC patients, these changes could not be confirmed in genetic analyses.

The next step in the analysis was to investigate the influence of the age of GC patients on the percentage of occurrence and expression level of TLR-2, -3, -4, and -9 receptors on CD3-CD56+ and CD3+CD56+ cells, with particular attention paid to the differences between the diffuse and intestinal types, which will allow for a better understanding of how age and histopathological type of tumor affect the immune response of patients. Detailed data are provided in Supplementary Materials Table S5. First, we showed differences between Diffuse ≤ 50 and Intestinal ≤ 50, which concerned changes in the expression of TLR-2 and TLR-3 on CD3-CD56+ cells, where patients with the diffuse type showed higher expression levels of TLR-2 (median 20.01) compared to the intestinal type (median 9.02, *p* = 0.009). Similarly, for TLR-3 on CD3-CD56+, diffuse patients had higher expression (median 10.74) compared to intestinal (median 4.70, *p* = 0.011). For TLR-4 and TLR-9 on CD3-CD56+, patients with diffuse ≤50 also showed higher expression than intestinal ≤ 50 (Supplementary Materials Table S5). Further, differences were shown between Diffuse ≤ 50 and Intestinal ≥ 51, where younger patients with the diffuse type (≤50) had higher expression levels of TLR-2, TLR-3, TLR-4, and TLR-9 on CD3-CD56+ cells compared to older patients with the intestinal type (≥51). For TLR-2, the median was 20.01 in the diffuse ≤ 50 groups compared to 6.00 in the intestinal ≥ 51 groups (*p* = 0.001), and similar differences were observed for TLR-3 (*p* = 0.002), TLR-4 (*p* = 0.000), and TLR-9 (*p* = 0.002), suggesting a more intense activation in the diffuse type in younger patients. Comparing Intestinal ≤ 50 with Diffuse ≥ 51, significantly higher expression levels of TLR-2, TLR-3, TLR-4, and TLR-9 were demonstrated in CD3-CD56+ cells in Diffuse ≥ 51 patients, which may suggest a more intense immune activation in the older age group in the diffuse type. In the comparison of Intestinal ≤ 50 with Intestinal ≥ 51, the differences were not widely evident. However, older patients with intestinal type showed slightly lower expression of TLR-2 on CD3-CD56+, which may suggest reduced immune activity with age. Finally, the comparative analysis between Diffuse ≥ 51 and Intestinal ≥ 51 showed higher expression levels of TLR-2, TLR-4, and TLR-9 on CD3-CD56+ and CD3+CD56+ cells in diffuse ≥ 51 patients (median TLR-2: 11.12 vs. 6.00, *p* = 0.008; TLR-4: 10.06 vs. 5.53, *p* = 0.000; TLR-9: 11.15 vs. 5.55, *p* = 0.013) (Supplementary Materials Table S5).

### 3.5. Analysis of Spearman’s Rank Correlation and ROC Curves for the Percentage of Occurrence and Expression Level of TLR-2, -3, -4, and -9 on CD3-CD56+ and CD3+CD56+

To better understand the relationship between the percentage of occurrence and the level of expression of TLR-2, -3, -4, and -9 receptors on CD3-CD56+ and CD3+CD56+ cells, Spearman rank correlation analysis was performed. ROC curves were assessed, which allowed us to assess the diagnostic ability of these parameters in the context of the immune response in patients. In the first analysis, we showed 338 statistically significant correlations for GC patients, among which 27.51% were negative correlations (63 low correlations, 29 moderate correlations, and one very high correlation), while the remaining 72.49% were positive correlations (35 low correlations, 32 moderate correlations, 130 high correlations, 35 very high correlations, and 13 total correlations). The results of the correlation analysis highlight several statistically significant associations between different immune parameters and TLR expression on CD3-CD56+ and CD3+CD56+ cells. Key findings include strong positive correlations between TLR-2, TLR-3, TLR-4, and TLR-9 expression on CD3-CD56+ cells, suggesting coordinated regulation of these receptors in response to immune challenges. Remarkably, TLR-2 expression on CD3-CD56+ cells showed one of the highest correlations with TLR-4 expression, suggesting a potentially synergistic role in modulating the immune response. Negative correlations were observed between the percentage of CD3+CD8+ T cells and the CD3+CD4+/CD3+CD8+ ratio, underscoring the inverse relationship between these T cell subpopulations in the context studied. Furthermore, stage-related correlations with TLR expression, particularly on CD3-CD56+ cells, underscore the progressive nature of immune changes in disease progression. These results provide insight into the dynamic interplay of immune cells and TLR expression, which may aid in developing targeted therapeutic strategies. Details are presented in Supplementary Material Table S6 and graphically in Supplementary Materials Figure S2.

ROC curve analyses were performed in three variants: the first concerned the analysis of the studied TLRs in the context of the disease stage in patients with GC; the second concerned the differences between the GC type with special consideration of HV; and the third concerned the changes in the individual age categories among patients with GC and HV. Detailed analyses are presented in Supplementary Materials Tables S7–S9.

In the first case, ROC curve analysis showed significant differences in the percentage of TLRs on CD3-CD56+ and CD3+CD56+ cells, which allows for effective differentiation of GC stages. For CD3-CD56+^dim^ TLR2+, TLR3+, TLR4+, and TLR9+, the AUC values were 1.0 for most comparisons, especially between later stages (III vs. IV), indicating excellent discrimination ability. Results for TLR2 + and TLR9 + in the comparison I vs. II achieved AUC of 0.74 (*p* = 0.0251) and 0.8267 (*p* = 0.0023), indicating significant, although slightly lower, diagnostic power in the early stages.

The percentage of TLR expression on CD3-CD56+^bright^ and CD3+CD56+ cells also showed high AUC values, especially for TLR2+ and TLR3+, where the AUC was 1.0 in most comparisons (*p* < 0.0001), highlighting their high specificity and sensitivity in differentiating GC stages. The results for CD3+CD56+TLR2+ and TLR9+ showed AUCs from 0.9 to 1.0, confirming their high discriminatory value, with significant results in all comparisons (*p* < 0.0001).

ROC curve analysis of TLR expression on CD3-CD56+ and CD3+CD56+ cells showed a high ability to differentiate GC stages, especially for TLR2, TLR3, and TLR9. TLR-2 and TLR-3 expression on CD3-CD56+ cells showed excellent diagnostic power in most comparisons with AUCs close to 1.0 (*p* < 0.0001), whereas TLR-4 and TLR-9 on CD3+CD56+ cells achieved high AUCs for later stages but slightly lower efficacy in early stages. Expression of TLR-2, TLR-3, TLR-4, and TLR-9 in the genetic material of CD3+CD56+ cells also showed variable but often high diagnostic power, especially in more advanced stages, with AUCs close to 1.0 in key comparisons (*p* < 0.0001) (Supplementary Materials Table S7).

In the second analyzed case, TLR expression on different CD3-CD56+ and CD3+CD56+ subtypes is particularly effective in differentiating GC patients from healthy individuals, with exceptionally high efficiency compared to the control group. The differentiation efficiency between Diffuse and Intestinal types was lower and did not always reach statistical significance. Among these parameters, the best differentiating are TLR expressions on CD3-CD56+ cells (TLR2+, TLR3+, TLR4+, TLR9+), which obtained the highest AUC values and statistical significance, suggesting their more excellent diagnostic value in distinguishing diffuse from intestinal type (Supplementary Materials Table S8).

In the third analyzed case, the analysis of AUC values showed that the parameters studied may be useful for differentiating GC patients below and above 50 years of age. Still, their diagnostic value is relatively moderate (Supplementary Materials Table S8). All observed changes in the studied parameters can effectively diagnose patients GC ≤ 50 with HV ≤ 50 and GC ≤ 50 with HV ≥ 51, as well as patients GC ≥ 51 with HV ≤ 50 and HV ≥ 51 (Supplementary Materials Table S8).

## 4. Discussion

Studies conducted by our team have shown significant differences in the expression of TLR receptors between groups of patients with GC and in comparison to the HV group, which may have important diagnostic and prognostic implications. The statistical analysis performed showed a significantly higher expression of the tested TLR receptors (TLR-2, TLR-3, TLR-4, and TLR-9) in the group of patients with GC compared to HV, which suggests that these receptors play an essential role in the immune response to GC. 

The signaling pathways involved in NK cell activation by TLRs differ depending on their location—TLR2 and TLR4 are usually present on the cell membrane surface, whereas TLR3 and TLR9 are located intracellularly and are activated upon entry of ligands into the cell. TLR2 recognizes lipoproteins and peptidoglycans derived from Gram-positive bacteria, as well as microbial and fungal components. TLR2 can form heterodimers with TLR1 or TLR6, which broadens the range of ligands it recognizes. Upon activation by a ligand, TLR2 recruits the adaptor MyD88 (myeloid differentiation primary response 88), which leads to the activation of the NF-κB pathway and, consequently, to the transcription of proinflammatory genes and the production of cytokines [6,7,8]. TLR4 is specific for lipopolysaccharides (LPS) present on the surface of Gram-negative bacteria. Its activation initiates a signaling cascade through both MyD88 and TRIF (Toll/IL-1 receptor domain-containing adaptor-inducing interferon-β), leading to the activation of two major signaling pathways: NF-κB and IRF3 (interferon regulatory factor 3). TLR4, in addition to NF-κB, stimulates the production of type I interferons, which enhances antiviral and antitumor responses [9,10,11]. TLR3 is located primarily in endosomes and recognizes double-stranded RNA (dsRNA), which is typical for many viruses. After binding dsRNA, TLR3 mainly activates the TRIF pathway, which leads to the production of proinflammatory cytokines, especially type I interferons. In NK cells, TLR3 activation increases their cytotoxicity and IFN-γ production, supporting antiviral and antitumor responses [12]. TLR9 is also intracellular and localized in endosomes, where it recognizes unmethylated CpG sequences found in the DNA of bacteria and some viruses. After activation, TLR9 uses the MyD88 adaptors, which leads to the activation of NF-κB and IRF7, resulting in the production of proinflammatory cytokines and interferons. In NK cells, TLR9 activation promotes their cytotoxic activity and cytokine production. Differences in TLR localization affect the way they detect pathogens and activate NK cells [13,14]. TLR2 and TLR4, as membrane receptors, enable a rapid response to external threats, while TLR3 and TLR9, as intracellular receptors, detect pathogens after they enter the cell, which is particularly important for antiviral responses and responses against intracellular pathogens.

One of the study’s key findings is the significantly higher expression of TLR in CD3-CD56+ and CD3+CD56+ cells in patients with GC compared to HV. The mean expression of TLR-2 in NK cells of patients with GC was 13.37±10.41, while in the HV group, it was only 1.24 ± 0.84 (*p* = 0.000). A similar pattern was observed for other receptors studied, where TLR-3, TLR-4, and TLR-9 were also significantly higher in the GC group. For example, the expression of TLR-9 in CD3-CD56+ cells was, on average, 12.42 ± 9.53 in the GC group and 1.26 ± 0.87 in the HV group (*p* = 0.000), which indicates apparent differences in the immune response between patients and healthy individuals. These results are consistent with the literature suggesting that TLRs may be vital in recognizing cancer cells and initiating the inflammatory response [15,16,17,18,19]. At the same time, the observed higher expression of TLR receptors in GC patients suggests that these receptors may be associated with disease progression. Studies show that TLR-2, TLR-3, TLR-4, and TLR-9 are involved in the immune response to tumors by stimulating CD3-CD56+ and CD3+CD56+cells, which may lead to increased cytotoxicity and production [20,21,22]. In the case of cancers such as GC, TLR expression may play a dual role: on the one hand, it may promote an anti-tumor response, and on the other hand, it may be associated with a chronic inflammatory state that supports tumor development [20,21,22]. Moreover, differences in TLR expression on CD3-CD56+ and CD3+CD56+cells may be related to adaptive changes in the immune response as the disease progresses. Data suggest that higher levels of TLR expression in mature NK cells are observed in more advanced stages of cancer, which may indicate an adaptive immune response of the organism to disease progression. In advanced stages of cancer, NK cells may increase TLR expression as an adaptive mechanism, enabling a more effective response to danger signals from the tumor microenvironment. It has been suggested that increased TLR expression may support NK cell activation and their ability to produce cytokines and destroy tumor cells, which is particularly important in the face of an increased number of tumor cells and the presence of pathogens or DNA fragments that stimulate TLRs. High TLR expression in mature NK cells, especially in those with the CD56dim phenotype, may indicate an increased readiness to respond to danger signals, which is crucial in antitumor and antiviral responses. NK cells with the CD56 dim phenotype are known for their high cytotoxicity—they are particularly effective in directly destroying cancer or virus-infected cells by releasing perforins and granzymes, which lead to apoptosis of target cells. As GC progresses, increased activation of TLRs may reflect an enhanced immune response, which in turn may impact tumor progression and patient prognosis. In studies of other cancers, such as skin cancer, TLR agonists have been successfully used to stimulate the immune response, suggesting that similar approaches may apply to the treatment of GC [14,18,23,24,25]. The differences in TLR expression between CD3-CD56+ and CD3+CD56+cells observed in our study may also provide additional information on the immunological mechanisms involved in gastric cancer. TLR expression was higher in patients with GC in both cell subpopulations, but in CD3-CD56+ cells, more significant differences were observed compared to healthy volunteers. This may indicate a more significant role of CD3-CD56+ cells in the antitumor response in gastric cancer. Moreover, increased TLR expression in CD3+CD56+ cells may suggest that these cells also play an essential role in the immune response to tumors. However, their role may be more related to the modulation of the inflammatory response than direct cytotoxicity [19,26,27]. 

It is also worth mentioning that the highest expression of TLRs was observed in patients in the advanced stages of the disease. This may indicate a more intense immune response activation as the disease progresses. In particular, the expression values of TLR-2, TLR-3, TLR-4, and TLR-9 on CD3-CD56+ cells increased proportionally with the tumor stage, suggesting that these receptors may be functional as diagnostic and prognostic biomarkers in assessing the progression of GC. Such a phenomenon may reflect the increasing demand for an immune response in more advanced stages of the disease [28,29]. Our studies suggest TLR receptors may become attractive therapeutic targets for treating GC. There are reports in the literature on the use of TLR agonists in treating other types of cancers, which indicates the possibility of using similar strategies in GC [14,26,27,28,30]. TLR9 agonists are currently being investigated in anticancer therapy, both as monotherapy and in combination with other treatments such as chemotherapy and immunotherapy. Moreover, increased expression of TLRs in CD3-CD56+ and CD3+CD56+ cells in GC patients suggests that stimulation of these receptors could contribute to an enhanced immune response to the tumor [14,26,27,28,30].

### Limitations of the Study

Although the results of the study presented in this publication are statistically significant, they only show a small fragment of the immunopathogenesis of GC. It should be noted that the study was conducted on a relatively small number of patients with GC and HV, which may limit the possibility of generalizing the results to a broader population. The study did not include a detailed analysis of potential confounding factors, such as lifestyle, diet, or exposure to environmental factors that may affect TLR expression in CD3-CD56+ and CD3+CD56+. Therefore, this aspect should also be analyzed in future studies. Moreover, the study participants differed not only in terms of the type of cancer (diffuse vs. intestinal), but also in the advancement of the disease. The insufficient number of these groups may limit the possibility of precise assessment of the relationship between TLR expression and specific clinical features of the patients. Additionally, the presented analyses were cross-sectional and did not include long-term monitoring of patients with GC, which makes it impossible to assess changes in TLR expression over time and their potential impact on disease progression, response to treatment, or prognosis. The obtained results of the study show us an insight into the deregulation of the immune system by CD3-CD56+ and CD3+CD56+ cells at the time of GC diagnosis and may be a good starting point for further detailed analyses. All these limitations indicate the need for further, more detailed studies with larger cohorts, taking into account the control of confounding factors and functional analysis of TLR receptors to better understand their role in the pathogenesis and potential treatment of GC.

## 5. Conclusions

The analysis of TLR expression on CD3-CD56+ and CD3+CD56+ cells in GC patients and healthy volunteers showed significant differences that could distinguish patients by stage, age, and cancer type. Increasing TLR expression with disease progression suggests an adaptive immune response in GC, particularly in advanced stages, reflecting intensified inflammation and immune activation that may affect clinical outcomes. TLR expression may serve as a biomarker for GC type and patient age, supporting diagnostic and personalized treatment approaches. Differences in TLR2, TLR3, TLR4, and TLR9 expression across immune cell subpopulations highlight the need for further research into the regulatory mechanisms of the immune response in GC. Future studies should focus on the molecular roles of individual TLRs at different GC stages, identifying potential therapeutic targets, and informing tailored immunotherapy strategies. Combining TLR expression data with other biomarkers could enhance diagnostic and therapeutic precision in GC treatment.

## Figures and Tables

**Table 1:** Summary of the obtained results of immunological tests for patients with newly diagnosed GC and healthy volunteers, with particular emphasis on the percentage of the tested TLRs on CD3-CD56+ and CD3+CD56+ cells.

Parameters	GC		HV		*p*-Value
	Mean ± SD	Median (Range)	Mean ± SD	Median (Range)	
Age	59.52 ± 13.13	59.00 (39.00–84.00)	58.65 ± 14.05	54.45 (42.00–87.00)	0.804
Gender	Females: 34.88% Males: 65.12%		Females: 43.33% Males: 56.67%		
CD45+ [%]	86.39 ± 11.03	89.53 (53.20–99.83)	88.02 ± 9.13	88.73 (55.28–99.18)	0.728
CD3-CD56+ [%]	11.10 ± 4.73	11.30 (3.05–25.19)	10.98 ± 2.83	11.64 (5.45–14.47)	0.923
CD3-CD56+^dim^ [%]	9.39 ± 4.23	9.10 (2.26–22.42)	9.21 ± 2.44	9.78 (4.36–12.82)	0.908
CD3-CD56+^bright^ [%]	1.47 ± 0.69	1.41 (0.23–3.43)	1.52 ± 0.62	1.32 (0.38–2.65)	0.627
CD3+CD56+ [%]	3.73 ± 2.59	3.13 (0.27–11.94)	1.84 ± 1.21	1.36 (0.29–4.27)	0.000 *
CD3+ [%]	67.58 ± 9.93	69.50 (39.88–88.04)	67.42 ± 8.11	67.62 (39.91–79.58)	0.756
CD19 B lymphocytes [%]	7.72 ± 3.72	6.79 (1.66–20.90)	9.62 ± 3.52	8.75 (4.50–17.59)	0.008 *
CD3+CD4+ T lymphocytes [%]	35.17 ± 10.68	34.49 (14.28–59.61)	37.62 ± 6.28	39.18 (20.39–49.18)	0.165
CD3+CD8+ T lymphocytes [%]	29.02 ± 8.78	29.55 (9.00–49.14)	27.96 ± 5.14	27.38 (18.64–38.72)	0.508
CD3+CD4+/CD3+CD8+ cells ratio	1.41 ± 0.79	1.23 (0.36–4.23)	1.40 ± 0.43	1.22 (0.86–2.62)	0.416
CD3-CD56+^dim^TLR-2+ [%]	10.18 ± 2.94	10.68 (4.04–14.99)	1.48 ± 0.64	1.30 (0.39–2.74)	0.000 *
CD3-CD56+^dim^TLR-3+ [%]	8.61 ± 3.13	8.98 (2.02–13.91)	0.69 ± 0.30	0.66 (0.22–1.53)	0.000 *
CD3-CD56+^dim^TLR-4+ [%]	10.26 ± 2.88	11.31 (4.25–13.99)	0.74 ± 0.37	0.69 (0.13–1.54)	0.000 *
CD3-CD56+^dim^TLR-9+ [%]	9.09 ± 3.07	9.70 (2.02–14.32)	0.90 ± 0.35	0.86 (0.29–1.68)	0.000 *
CD3-CD56+b^right^TLR-2+ [%]	8.18 ± 2.91	8.15 (2.71–15.22)	0.56 ± 0.28	0.48 (0.12–1.29)	0.000 *
CD3-CD56+^bright^TLR-3+ [%]	7.00 ± 2.98	7.62 (1.35–14.08)	0.54 ± 0.22	0.51 (0.18–1.13)	0.000 *
CD3-CD56+^bright^TLR-4+ [%]	8.28 ± 2.90	8.84 (2.85–13.88)	0.47 ± 0.23	0.46 (0.07–0.97)	0.000 *
CD3-CD56+^bright^TLR-9+ [%]	7.34 ± 2.92	7.44 (1.35–14.67)	0.57 ± 0.23	0.56 (0.16–1.21)	0.000 *
CD3+CD56+TLR-2+ [%]	8.07 ± 2.78	8.37 (3.00–12.97)	0.85 ± 0.34	0.81 (0.28–1.60)	0.000 *
CD3+CD56+TLR-3+ [%]	7.41 ± 2.60	7.43 (3.07–13.85)	0.37 ± 0.27	0.23 (0.06–0.94)	0.000 *
CD3+CD56+TLR-4+ [%]	7.97 ± 2.39	8.12 (3.30–13.46)	0.79 ± 0.42	0.66 (0.28–2.05)	0.000 *
CD3+CD56+TLR-9+ [%]	7.18 ± 2.84	7.18 (2.01–12.91)	0.46 ± 0.35	0.30 (0.07–1.26)	0.000 *

* statistically significant results.

**Table 2:** Evaluation of the expression of the studied TLR receptors in the genetic material derived from CD3-CD56+ to CD3+CD56+ subpopulations sorted from PBMC between the GC and HV groups.

Parameters	GC		HV		*p*-Value
	Mean ± SD	Median (Range)	Mean ± SD	Median (Range)	
TLR-2 expression in CD3-CD56+ cells	13.37 ± 10.41	10.85 (0.25–40.09)	1.24 ± 0.84	1.06 (0.31–3.69)	0.000 *
TLR-3 expression in CD3-CD56+ cells	7.65 ± 5.50	5.83 (0.58–22.62)	1.26 ± 0.87	1.06 (0.31–3.77)	0.000 *
TLR-4 expression in CD3-CD56+ cells	12.31 ± 8.99	10.50 (0.34–45.11)	1.27 ± 0.90	1.05 (0.31–3.75)	0.000 *
TLR-9 expression in CD3-CD56+ cells	12.42 ± 9.53	9.77 (0.10–41.83)	1.26 ± 0.87	1.06 (0.31–3.79)	0.000 *
TLR-2 expression in CD3+CD56+ cells	6.78 ± 7.35	3.72 (0.28–43.56)	1.30 ± 0.94	0.92 (0.28–3.71)	0.000 *
TLR-3 expression in CD3+CD56+ cells	7.38 ± 4.93	5.61 (0.55–24.04)	1.38 ± 1.12	0.94 (0.16–4.91)	0.000 *
TLR-4 expression in CD3+CD56+ cells	5.51 ± 4.57	4.30 (0.11–20.50)	1.31 ± 0.98	1.02 (0.23–4.66)	0.000 *
TLR-9 expression in CD3+CD56+ cells	8.19 ± 5.31	7.31 (0.38–22.86)	1.21 ± 0.80	0.94 (0.24–4.21)	0.000 *

* statistically significant results.

## Data Availability

All data are available on written request from the first author of this publication.

## Supplementary Materials

The following supporting information can be downloaded at: https://www.mdpi.com/article/10.3390/cancers16223854/s1, Table S1. Selected results of peripheral blood morphology and biochemistry in patients with GC and HV; Table S2. The influence of disease stage on the percentage of occurrence and expression level of TLR-2, -3, -4, and -9 on NK and NKT-like cells in patients with GC; Table S3. The influence of GC type on the percentage of occurrence and expression level of TLR-2, -3, -4, and -9 on NK and NKT-like cells in patients with GC vs. HV; Table S4. The influence of patient age on the percentage of occurrence and expression level of TLR-2, -3, -4, and -9 on NK and NKT-like cells; Table S5. The influence of age of GC patients on the percentage of occurrence and expression level of TLR-2, -3, -4, and -9 on NK and NKT-like cells with particular emphasis on differences between the diffuse and intestinal type; Table S6. Spearman rank correlations; Table S7. Tabulated summary of ROC curves for disease stage among GC patients; Table S8. Tabulated summary of ROC curves for diffuse and intestinal types of GC and HV; Table S9. Tabulated summary of ROC curves for age categories of patients with GC and HV; Figure S1: Example of flow cytometric analysis; Figure S2. Schematic representation of Spearman rank correlations for individual clinical and immunological parameters of newly diagnosed GC patients.
