# Supplementary material for: Can the Analysis of Toll-like Receptors (TLR) on NK and NKT-like Cells Improve Gastric Cancer Diagnostics and Treatment?
Source: Cancers (Basel). 2024 Nov 17;16(22):3854. doi: 10.3390/cancers16223854 (PMC11592653; doi:10.3390/cancers16223854)
Supplement: Supplementary file 1 [file cancers-16-03854-s001.zip › cancers-3264308-supplementary.pdf]

Supplementary Materials Table S1- Selected results of peripheral blood morphology and biochemistry in patients with GC and HV.

Parameters	GC		HV		p-Value
	Mean±SD	Median (Range)	Mean±SD	Median (Range)	
White blood cells [10^3/mm^3]	7.47±2.01	7.34 (4.12-10.88)	5.90±0.92	5.97 (4.13-7.81)	0.000*
Neutrophils [10^3/mm^3]	4.24±1.05	4.02 (2.20-6.10)	4.91±1.03	5.22 (2.77-6.10)	0.004*
Monocytes [10^3/mm^3]	0.46±0.12	0.47 (0.09-0.75)	0.49±0.11	0.52 (0.32-0.68)	0.259
Lymphocytes [10^3/mm^3]	2.34±0.56	2.31 (1.37-4.10)	2.25±0.50	2.27 (1.47-3.14)	0.566
PLT [10^3/mm^3]	176.43±50.10	173.94 (91.35-267.00)	263.93±56.46	255.50 (190.00-358.00)	0.000*
HGB [g/dl]	11.89±1.74	11.69 (8.78-14.90)	16.13±1.15	16.29 (14.32-17.97)	0.000*
RBC [10^6/mm^3]	3.27±0.83	3.20 (1.76-4.99)	4.03±0.54	3.98 (3.14-4.97)	0.000*

Supplementary materials Tabel S2. The influence of disease stage on the prevalence and expression level of TLR-2, -3, -4 and -9 on NK and NKT-like cells in GC patients.

Parameters	Stage I		Stage II		Stage III		Stage IV		p-Value					
	Mean±SD	Median (Range)	Mean±SD	Median (Range)	Mean±SD	Median (Range)	Mean±SD	Median (Range)	I vs. II	I vs. III	I vs. IV	II vs. III	II vs. IV	III vs. IV
**Age**	61.60±10.81	63.00 (46.00-78.00)	63.07±14.32	62.00 (41.00-84.00)	58.25±13.39	58.50 (39.00-82.00)	57.60±12.54	57.50 (41.00-79.00)	0.775	0.362	0.330	0.221	0.227	0.966
**CD45+ [%]**	85.82±7.01	85.91 (71.55-94.18)	90.00±9.20	92.70 (61.25-98.25)	85.72±12.63	90.63 (54.71-99.83)	85.32±11.10	87.78 (53.20-99.38)	0.050	0.351	0.730	0.373	0.139	0.525
**NK cells [%]**	15.45±2.81	15.86 (10.22-20.62)	11.73±3.72	11.67 (6.27-18.32)	10.83±5.04	9.78 (3.05-25.19)	7.84±2.99	7.94 (3.09-13.71)	0.008 *	0.001*	0.000*	0.546	0.004*	0.034*
**CD3-CD56+^{dim} [%]**	13.46±2.47	13.48 (9.10-17.53)	10.06±3.54	9.35 (5.02-16.30)	9.10±4.41	7.92 (2.26-22.42)	6.35±2.42	6.43 (2.50-11.11)	0.008 *	0.000*	0.000*	0.407	0.004*	0.022*
**CD3-CD56+^{bright} [%]**	1.75±0.48	1.73	1.44±0.44	1.41	1.49±0.85	1.22	1.25±0.57	1.27	0.098	0.088	0.012*	0.767	0.254	0.514

		(0.88-2.85)		(0.58-2.10)		(0.23-3.43)		(0.35-2.36)						
**CD3+CD56+ [%]**	6.74±1.96	6.70 (3.39-9.94)	2.85±2.03	2.12 (0.61-7.22)	3.27±2.31	3.05 (0.27-11.94)	2.97±2.23	1.96 (0.71-8.13)	0.000*	0.000*	0.000*	0.574	0.882	0.629
**CD3+ [%]**	62.02±5.36	61.61 (51.27-69.66)	68.65±9.96	69.68 (39.88-81.63)	67.94±10.14	70.53 (45.54-88.04)	70.30±10.55	72.89 (40.25-82.92)	0.008*	0.028*	0.001*	0.846	0.330	0.304
**CD19 B lymphocytes [%]**	8.35±2.48	7.56 (5.49-15.23)	9.61±4.88	9.77 (1.88-20.90)	6.96±3.53	6.04 (1.66-16.48)	7.18±3.21	5.95 (2.76-14.35)	0.412	0.033*	0.099	0.070	0.179	0.966
**CD3+CD4+ T lymphocytes [%]**	32.06±4.40	32.63 (24.19-40.01)	37.31±8.84	35.27 (21.35-57.21)	34.38±11.42	32.63 (14.60-59.61)	37.35±12.87	38.36 (14.28-57.40)	0.098	0.690	0.158	0.373	0.831	0.391
**CD3+CD8+ T lymphocytes [%]**	25.11±9.16	23.82 (9.00-40.26)	28.58±9.78	29.09 (11.79-48.07)	30.29±7.39	29.92 (15.37-48.70)	29.99±9.14	29.28 (16.77-49.14)	0.367	0.101	0.202	0.616	0.780	0.832
**CD3+CD4+/CD3+CD8+ cells ratio**	1.54±0.80	1.39 (0.60-3.70)	1.56±0.94	1.21 (0.64-4.23)	1.25±0.63	1.08 (0.36-2.89)	1.46±0.86	1.31 (0.38-3.18)	0.870	0.281	0.681	0.281	0.633	0.547
**CD3-CD56+^{dim}TLR-2+ [%]**	6.20±1.14	6.05 (4.04-7.85)	7.42±1.46	7.72 (5.07-9.66)	11.35±1.61	11.19 (9.05-13.98)	13.11±1.15	12.87 (11.16-14.99)	0.023*	0.000*	0.000*	0.000*	0.000*	0.000*
**CD3-CD56+^{dim}TLR-3+ [%]**	3.26±1.12	2.78 (2.02-5.28)	7.11±1.02	7.22 (5.62-8.83)	9.52±1.02	9.23 (8.01-11.92)	12.12±1.26	12.09 (10.08-13.91)	0.000*	0.000*	0.000*	0.000*	0.000*	0.000*
**CD3-CD56+^{dim}TLR-4+ [%]**	5.73±0.91	5.85 (4.25-7.07)	7.81±1.25	7.52 (5.27-9.69)	11.90±1.31	12.09 (9.47-13.99)	12.54±0.85	12.67 (11.16-13.91)	0.000*	0.000*	0.000*	0.000*	0.000*	0.096
**CD3-CD56+^{dim}TLR-9+ [%]**	4.48±1.64	4.30 (2.02-6.47)	6.68±1.25	6.44 (5.03-8.99)	10.36±1.40	10.43 (8.22-12.47)	12.05±1.37	12.00 (9.82-14.32)	0.002*	0.000*	0.000*	0.000*	0.000*	0.000*
**CD3-CD56+^{bright}TLR-2+ [%]**	4.34±0.72	4.62 (2.71-5.26)	5.76±1.22	5.43 (3.75-7.59)	9.17±1.73	8.61 (5.83-12.43)	11.09±1.97	10.83 (8.15-15.22)	0.001*	0.000*	0.000*	0.000*	0.000*	0.001*
**CD3-CD56+^{bright}TLR-3+ [%]**	2.32±0.87	2.06 (1.35-4.07)	5.53±0.97	5.63 (3.79-7.01)	7.77±1.60	7.99 (5.05-10.61)	10.22±1.70	9.79 (7.96-14.08)	0.000*	0.000*	0.000*	0.000*	0.000*	0.000*
**CD3-CD56+^{bright}TLR-4+ [%]**	4.04±0.72	4.29 (2.85-5.44)	6.08±1.20	5.60 (3.90-7.85)	9.70±1.95	10.32 (5.97-12.42)	10.55±1.38	10.14 (8.36-13.88)	0.000*	0.000*	0.000*	0.000*	0.000*	0.211
**CD3-CD56+^{bright}TLR-9+ [%]**	3.16±1.18	3.31 (1.35-4.93)	5.21±1.20	4.77 (3.41-7.55)	8.39±1.58	7.86 (5.50-11.08)	10.18±1.85	10.15 (7.17-14.67)	0.000*	0.000*	0.000*	0.000*	0.000*	0.001*
**CD3+CD56+TLR-2+ [%]**	4.09±0.82	4.06 (3.00-5.67	5.74±0.79	5.79 (4.67-6.95	8.83±1.02	8.74 (7.02-10.79)	11.43±1.45	11.90 (8.31-12.97	0.000*	0.000*	0.000*	0.000*	0.000*	0.000*
**CD3+CD56+TLR-3+ [%]**	3.77±0.58	3.63 (3.07-4.78)	6.58±1.68	6.77 (4.26-8.84)	7.43±0.89	7.46 (6.03-8.95)	10.73±1.94	11.06 (7.21-13.85)	0.000*	0.000*	0.000*	0.165	0.000*	0.000*
**CD3+CD56+TLR-4+ [%]**	5.01±1.27	4.91 (3.30-6.85)	5.86±0.78	5.90 (4.29-7.33)	8.49±0.89	8.39 (7.07-9.97)	10.85±1.68	10.63 (8.61-13.46)	0.098	0.000*	0.000*	0.000*	0.000*	0.000*
**CD3+CD56+TLR-9+ [%]**	2.79±0.50	2.82 (2.01-3.70)	5.58±0.82	5.56 (4.31-7.64)	7.72±1.21	7.51 (6.03-9.69)	10.70±1.46	11.04 (7.96-12.91)	0.000*	0.000*	0.000*	0.000*	0.000*	0.000*

**TLR-2 expression in NK cells**	1.69±0.95	1.29 (0.25-3.43)	5.27±1.78	4.81 (2.64-8.40)	14.10±6.21	12.80 (5.21-34.18)	26.90±7.64	27.50 (11.84-40.09)	0.000*	0.000*	0.000*	0.000*	0.000*	0.000*
**TLR-3 expression in NK cells**	2.05±1.14	2.15 (0.58-4.79)	4.17±1.44	3.96 (2.00-6.58)	7.55±3.84	6.69 (2.67-20.91)	14.62±4.47	15.00 (6.10-22.62)	0.002*	0.000*	0.000*	0.000*	0.000*	0.000*
**TLR-4 expression in NK cells**	2.47±1.32	2.11 (0.34-5.27)	5.59±4.59	4.33 (2.04-21.80)	15.76±8.32	13.09 (5.62-45.11)	18.50±6.30	18.77 (6.98-32.01)	0.001*	0.000*	0.000*	0.000*	0.000*	0.145
**TLR-9 expression in NK cells**	2.52±1.88	2.07 (0.10-6.26)	4.49±1.69	4.64 (2.08-7.55)	13.34±6.06±	12.09 (4.62-30.49)	24.16±7.73	22.63 (9.46-41.83)	0.019*	0.000*	0.000*	0.000*	0.000*	0.000*
**TLR-2 expression in NKT cells**	1.43±0.77	1.38 (0.28-2.68)	1.45±0.88	1.21 (0.51-.07)	5.27±2.98	4.12 (1.41-13.09)	17.49±7.35	16.21 (6.52-43.56)	0.512	0.000*	0.000*	0.000*	0.000*	0.000*
**TLR-3 expression in NKT cells**	3.03±1.62	2.85 (0.55-5.46)	6.39±3.88	5.35 (2.53-18.79)	8.80±5.46	7.55 (2.30-24.04)	8.82±4.13	8.95 (2.67-18.64)	0.000*	0.000*	0.000*	0.096	0.055	0.716
**TLR-4 expression in NKT cells**	1.92±0.93	2.10 (0.33-3.91)	3.23±2.39	2.51 (0.11-7.56)	6.09±4.84	4.71 (0.92-20.50)	8.87±4.16	9.85 (2.25-14.98)	0.126	0.000*	0.000*	0.030*	0.000*	0.042*
**TLR-9 expression in NKT cells**	2.16±1.77	1.54 (0.38-6.91)	4.61±1.88	4.84 (2.31-9.26)	8.84±3.67	8.27 (2.96-18.44)	14.25±4.16	14.56 (4.98-22.86)	0.000*	0.000*	0.000*	0.000*	0.000*	0.021*

Supplementary materials Tabel S3. The influence of GC type on the percentage of occurrence and expression level of TLR-2, -3, -4 and -9 on NK and NKT-like cells in patients with GC versus HV.

**Parameters**	**Diffuse**		**Intestinal**		**HV**		**p-Value**		
	**Mean±SD**	**Median (Range)**	**Mean±SD**	**Median (Range)**	**Mean±SD**	**Median (Range)**	**D vs. I**	**D vs. HV**	**I vs. HV**
**Age**	60.35±13.26	58.00 (41.00-83.00)	58.31±12.83	61.00 (39.00-84.00)	58.65±14.05	54.45 (42.00-87.00)	0.446	0.610	0.870
**CD45+ [%]**	84.55±12.30	88.07 (53.20-99.38)	89.08±8.12	91.73 (69.59-99.83)	88.02±9.13	88.73 (55.28-99.18)	0.136	0.428	0.729
**NK cells [%]**	10.10±4.40	9.71 (3.05-18.51)	12.56±4.81	12.07 (4.23-25.19)	10.98±2.83	11.64 (5.45-14.47)	0.022*	0.252	0.179
**CD3-CD56+^{dim}[%]**	8.48±3.93	7.87 (2.26-16.47)	10.71±4.31	10.59 (3.76-22.42)	9.21±2.44	9.78 (4.36-12.82)	0.024*	0.203	0.143

**CD3-CD56+^{bright} [%]**	1.38±0.66	1.32 (0.35-3.05)	1.61±0.71	1.57 (0.23-3.43)	1.52±0.62	1.32 (0.38-2.65)	0.088	0.277	0.661
**CD3+CD56+ [%]**	3.52±2.29	3.16 (0.61-8.95)	4.04±2.95	3.09 (0.27-11.94)	1.84±1.21	1.36 (0.29-4.27)	0.612	0.001*	0.001*
**CD3+ [%]**	66.93±10.77	68.73 (39.88-88.04)	68.52±8.47	70.30 (53.49-82.85)	67.42±8.11	67.62 (39.91-79.58)	0.594	0.873	0.671
**CD19 B lymphocytes [%]**	7.52±3.70	6.65 (1.66-20.90	8.01±3.73	6.79 (2.91-16.48)	9.62±3.52	8.75 (4.50-17.59)	0.637	0.013*	0.028*
**CD3+CD4+ T lymphocytes [%]**	34.70±10.57	33.77 (16.25-59.61)	35.86±10.80	36.56 (14.28-57.21)	37.62±6.28	39.18 (20.39-49.18)	0.546	0.077	0.606
**CD3+CD8+ T lymphocytes [%]**	28.74±8.93	29.55 (9.00-49.14)	29.42±8.56	29.55 (11.79-48.70)	27.96±5.14	27.38 (18.64-38.72)	0.834	0.637	0.460
**CD3+CD4+/CD3+CD8+ cells ratio**	1.40±0.78	1.11 (0.43-3.70)	1.41±0.80	1.42 (0.36-4.23)	1.40±0.43	1.22 (0.86-2.62)	0.841	0.190	0.953
**CD3-CD56+^{dim}TLR-2+ [%]**	10.85±2.84	11.16 (4.04-14.99)	9.19±2.81	9.17 (4.78-13.73)	1.48±0.64	1.30 (0.39-2.74)	0.009*	0.000*	0.000*
**CD3-CD56+^{dim}TLR-3+ [%]**	9.02±3.02	9.08 (2.13-13.91)	8.03±3.20	8.62 (2.02-13.67)	0.69±0.30	0.66 (0.22-1.53)	0.143	0.000*	0.000*
**CD3-CD56+^{dim}TLR-4+ [%]**	10.63±2.77	11.43 (4.25-13.96)	9.73±2.94	9.64 (4.39-13.99)	0.74±0.37	0.69 (0.13-1.54)	0.223	0.000*	0.000*
**CD3-CD56+^{dim}TLR-9+ [%]**	9.56±2.98	10.23 (2.02-14.32)	8.40±3.08	8.67 (2.44-13.68)	0.90±0.35	0.86 (0.29-1.68)	0.093	0.000*	0.000*
**CD3-CD56+^{bright}TLR-2+ [%]**	8.82±2.88	8.61 (2.71-15.22)	7.25±2.69	6.97 (3.68-13.18)	0.56±0.28	0.48 (0.12-1.29)	0.013*	0.000*	0.000*
**CD3-CD56+^{bright}TLR-3+ [%]**	7.41±2.92	7.92 (1.43-12.97)	6.41±2.97	6.20 (1.35-14.08)	0.54±0.22	0.51 (0.18-1.13)	0.143	0.000*	0.000*
**CD3-CD56+^{bright}TLR-4+ [%]**	8.68±2.88	9.50 (2.85-13.88)	7.70±2.83	7.82 (2.94-11.93)	0.47±0.23	0.46 (0.07-0.97)	0.145	0.000*	0.000*
**CD3-CD56+^{bright}TLR-9+ [%]**	7.82±2.90	7.86 (1.35-14.67)	6.63±2.79	7.19 (1.88-12.12)	0.57±0.23	0.56 (0.16-1.21)	0.061	0.000*	0.000*
**CD3+CD56+TLR-2+ [%]**	8.48±2.85	8.64	7.48±2.56	7.89	0.85±0.34	0.81	0.108	0.000*	0.000*

		(3.00-12.97)		(3.02-12.53)		(0.28-1.60)			
**CD3-CD3+CD56+TLR-3+ [%]**	7.65±2.52	7.45 (3.21-13.72)	7.06±2.67	7.37 (3.07-13.85)	0.37±0.27	0.23 (0.06-0.94)	0.314	0.000*	0.000*
**CD3-CD3+CD56+TLR-4+ [%]**	8.31±2.43	8.34 (3.45-13.46)	7.48±2.24	7.89 (3.30-12.65)	0.79±0.42	0.66 (0.28-2.05)	0.108	0.000*	0.000*
**CD3-CD3+CD56+TLR-9+ [%]**	7.73±2.93	7.96 (2.09-12.91)	6.37±2.50	6.40 (2.01-11.91)	0.46± 0.35	0.30 (0.07-1.26)	0.031*	0.000*	0.000*
**TLR-2 expression in NK cells**	16.79±11.38	16.68 (0.97-40.09)	8.39±6.00	8.51 (0.25-26.35)	1.24±0.84	1.06 (0.31-3.69)	0.001*	0.000*	0.000*
**TLR-3 expression in NK cells**	9.47±6.08	8.80 (0.75-22.62)	5.00±2.94	4.79 (0.58-15.54)	1.26±.87	1.06 (0.31-3.77)	0.001*	0.000*	0.000*
**TLR-4 expression in NK cells**	15.36±9.79	14.74 (1.51-45.11)	7.86±5.08	7.99 (0.34-18.65)	1.27±0.90	1.05 (0.31-3.75)	0.000*	0.000*	0.000*
**TLR-9 expression in NK cells**	15.42±10.53	15.85 (0.10-41.83)	8.05±5.42	7.49 (1.23-22.66)	1.26±0.87	1.06 (0.31-3.79)	0.001*	0.000*	0.000*
**TLR-2 expression in NKT cells**	8.32±8.53	4.07 (0.69-43.56)	4.53±4.28	2.68 (0.28-16.80)	1.30±0.94	0.92 (0.28-3.71)	0.032*	0.000*	0.000*
**TLR-3 expression in NKT cells**	7.56±5.06	5.46 (1.43-24.04)	7.11±4.74	5.75 (0.55-21.31)	1.38±1.12	0.94 (0.16-4.91)	0.807	0.000*	0.000*
**TLR-4 expression in NKT cells**	5.87±4.83	4.32 (0.20-20.50)	4.99±4.11	4.28 (0.11-17.39)	1.31±0.98	1.02 (0.23-4.66)	0.546	0.000*	0.000*
**TLR-9 expression in NKT cells**	9.15±5.59	7.97 (0.38-22.86)	6.81±4.52	5.66 (0.40-19.56)	1.21±0.80	0.94 (0.24-4.21)	0.067	0.000*	0.000*

Supplementary materials Tabel S4. The influence of patient age on the percentage of occurrence and expression level of TLR-2, -3, -4 and -9 on NK and NKT-like cells

**Parameters**	**GC ≤50**		**GC ≥51**		**HV ≤50**		**HV ≥51**		**p-Value**					
	**Mean±SD**	**Median (Range)**	**Mean±SD**	**Median (Range)**	**Mean±SD**	**Median (Range)**	**Mean±SD**	**Median (Range)**	**I vs. II**	**I vs. III**	**I vs. IV**	**II vs. III**	**II vs. IV**	**III vs. IV**
**Age**	45.36±3.15	46.00 (39.00-50.00)	68.34±8.42	68.00 (52.00-4.00)	46.04±3.04	45.24 (42.00-50.12)	67.06± 12.08	64.61 (50.82-87.00)	0.000*	0.502	0.000*	0.000*	0.551	0.000*

**CD45+ [%]**	87.42±11.01	89.17 (54.71-99.83)	85.75±10.99	89.89 (53.20-98.49)	88.33±6.04	88.25 (75.89-99.18)	87.81±0.70	89.69 (55.28-99.00)	0.452	0.830	0.853	0.887	0.551	0.884
**NK cells [%]**	11.01±4.62	9.78 (3.69-25.19)	11.15±4.80	11.65 (3.05-20.62)	10.87±2.67	11.73 (5.61-13.96)	11.05±2.92	11.57 (5.45-14.47)	0.818	0.713	0.632	0.874	0.860	0.819
**CD3-CD56+^{dim} [%]**	9.32±4.14	7.92 (2.99-22.42)	9.43±4.29	9.78 (2.26-7.53)	9.24±2.33	9.87 (4.99-12.42)	9.20±2.51	9.70 (4.36-12.82)	0.874	0.695	0.689	0.927	0.880	0.950
**CD3-CD56+^{bright}[%]**	1.44±0.65	1.25 (0.46-3.05)	1.49±.71	1.41 (0.23-3.43)	1.39±0.57	1.27 (0.38-2.34)	1.61±0.64	1.43 (0.61-2.65)	0.916	0.990	0.452	0.822	0.499	0.545
**CD3+CD56+ [%]**	3.11±2.22	2.47 (0.27-7.22)	4.12±2.73	3.39 (0.53-11.94)	1.75±0.81	1.40 (0.84-3.68)	1.90±1.41	1.24 (0.29-4.27)	0.083	0.164	0.045*	0.003*	0.001*	0.602
**CD3+ [%]**	68.86±9.43	70.67 (45.54-82.85)	66.78±10.15	67.45 (39.88-88.04)	68.10±5.53	67.62 (59.53-79.58)	66.97±9.42	67.62 (39.91-79.58)	0.331	0.354	0.440	0.848	0.849	0.884
**CD19 B lymphocytes [%]**	7.55±3.48	6.65 (1.66-16.48)	7.82±3.86	7.01 (1.88-20.90)	9.36±3.69	8.24 (4.50-17.59)	9.79±3.40	9.34 (4.50-17.59)	0.805	0.122	0.021*	0.176	0.031*	0.755
**CD3+CD4+ T lymphocytes [%]**	35.76±10.99	35.27 (14.60-57.40)	34.81±10.46	33.32 (14.28-59.61)	37.93±6.47	37.42 (29.17-49.18)	37.41±6.15	39.18 (20.39-48.84	0.659	0.534	0.618	0.243	0.190	0.950
**CD3+CD8+ T lymphocytes [%]**	30.03±7.04	30.19 (15.37-49.14)	28.39±.66	28.38 (9.00-48.70)	28.42±3.89	27.95 (18.64-33.75)	27.66±5.81	27.38 (18.64-38.72)	0.377	0.395	0.236	0.887	0.798	0.662
**CD3+CD4+/CD3+CD8+ cells ratio**	1.30±0.61	1.21 (0.36-2.95)	1.47± 0.88	1.25 (0.38-4.23)	1.39±0.45	1.24 (0.86-2.62)	1.41±0.41	1.21 (1.01-2.62)	0.784	0.676	0.417	0.707	0.533	0.917
**CD3-CD56+^{dim}TLR-2+ [%]**	10.57±3.11	11.16 (4.04-14.99)	9.93±2.80	9.66 (4.76-14.78)	1.75±0.64	1.94 (0.79-2.74)	1.30±0.58	1.04 (0.39-2.60)	0.305	0.000*	0.000*	0.000*	0.000*	0.039*
**CD3-CD56+^{dim}TLR-3+ [%]**	8.98±2.74	9.54 (2.13-13.91)	8.39±3.33	8.51 (2.02-13.88)	0.63±0.31	0.55 (0.22-1.40)	0.72±0.29	0.68 (0.22-1.53)	0.344	0.000*	0.000*	0.000*	0.000*	0.249
**CD3-CD56+^{dim}TLR-4+ [%]**	10.97±2.51	11.88 (4.87-13.91)	9.82±3.00	10.19 (4.25-13.99)	0.67±0.46	0.53 (0.13-1.54)	0.78±0.28	0.78 (0.29-1.40)	0.118	0.000*	0.000*	0.000*	0.000*	0.249
**CD3-CD56+^{dim}TLR-9+ [%]**	9.86±3.09	10.27 (2.52-14.24)	8.60±2.96	8.73 (2.02-14.32)	1.01±0.28	1.00 (0.65-1.68)	0.82±0.38	0.77 (0.29-1.68)	0.037*	0.000*	0.000*	0.000*	0.000*	0.185
**CD3-CD56+^{bright}TLR-2+ [%]**	8.69±2.94	9.27 (2.71-13.07)	7.86±2.85	7.74 (3.19-15.22)	0.59±0.31	0.53 (0.12-1.29)	0.54±0.25	0.44 (0.12-1.04)	0.167	0.000*	0.000*	0.000*	0.000*	0.632
**CD3-CD56+^{bright}TLR-3+ [%]**	7.42±2.58	8.06 (1.43-11.96)	6.74±3.19	6.20 (1.35-14.08)	0.50±0.24	0.43 (0.18-1.04)	0.56±0.21	0.55 (0.18-1.13)	0.313	0.000*	0.000*	0.000*	0.000*	0.285
**CD3-CD56+^{bright}TLR-4+ [%]**	9.01±2.59	9.60 (3.75-13.88)	7.82±2.99	7.65 (2.85-12.52)	0.44±0.29	0.35 (0.07-0.97)	0.49±0.17	0.47 (0.21-0.88)	0.118	0.000*	0.000*	0.000*	0.000*	0.325
**CD3-CD56+^{bright}TLR-9+ [%]**	8.14±2.95	8.75 (1.69-14.67)	6.83±2.78	7.23 (1.35-12.82)	0.65±0.22	0.62 (0.35-1.21)	0.51±0.22	0.46 (0.16-0.96)	0.029*	0.000*	0.000*	0.000*	0.000*	0.124
**CD3-CD3+CD56+TLR-2+ [%]**	8.68±2.55	8.64	7.69±2.85	8.16	0.96±0.27	0.95	0.78±0.36	0.73	0.125	0.000*	0.000*	0.000*	0.000*	0.185

		(3.00-12.91)		(3.02-12.97)		(0.62-1.60)		(0.28-1.60)						
**CD3-CD3+CD56+TLR-3+ [%]**	7.82±2.66	7.67 (3.08-13.85)	7.15±2.53	7.33 (3.07-13.72)	0.29±0.22	0.22 (0.06-0.68)	0.42±0.29	0.35 (0.11-0.94)	0.296	0.000*	0.000*	0.000*	0.000*	0.185
**CD3-CD3+CD56+TLR-4+ [%]**	8.41±2.36	8.61 (3.74-13.13)	7.70±2.37	7.76 (3.30-13.46)	0.71±0.35	0.62 (0.28-1.63)	0.85±0.45	0.66 (0.28-2.05)	0.129	0.000*	0.000*	0.000*	0.000*	0.439
**CD3-CD3+CD56+TLR-9+ [%]**	7.46±2.81	7.06 (2.09-12.10)	7.00±2.85	7.30 (2.01-12.91)	0.36±0.29	0.27 (0.07-.91)	0.52±0.37	0.41 (0.15-1.26)	0.566	0.000*	0.000*	0.000*	0.000*	0.200
**TLR-2 expression in NK cells**	14.88±10.46	13.13 (0.97-40.09)	12.43±10.28	9.19 (0.25-37.66)	1.24±0.70	1.14 (0.34-2.90)	1.25±0.92	1.00 (0.31-3.69)	0.243	0.000*	0.000*	0.000*	0.000*	0.755
**TLR-3 expression in NK cells**	8.20±5.29	7.45 (0.75-20.55)	7.31±.60	5.65 (0.58-22.62)	1.24±0.72	1.12 (0.32-3.03)	1.26±0.95	1.01 (0.31-3.77)	0.313	0.000*	0.000*	0.000*	0.000*	0.755
**TLR-4 expression in NK cells**	13.92±8.32	12.55 (1.51-32.01)	11.31±9.25	8.61 (0.34-45.11)	1.26±0.77	1.12 (0.31-3.22)	1.27±0.97	1.03 (0.32-3.75)	0.112	0.000*	0.000*	0.000*	0.000*	0.787
**TLR-9 expression in NK cells**	13.45±8.67	13.05 (0.10-30.13)	11.78± 9.98	8.39 (0.38-41.83)	1.25±0.72	1.13 (0.33-3.01)	1.27±0.95	1.01 (0.31-3.79)	0.269	0.000*	0.000*	0.000*	0.000*	0.755
**TLR-2 expression in NKT cells**	7.67±8.59	4.07 (0.51-43.56)	6.22±6.40	3.33 (0.28-23.10)	0.98±0.89	0.71 (0.32-3.71)	1.51±0.90	1.34 (0.28-3.31)	0.243	0.000*	0.000*	0.000*	0.000*	0.065
**TLR-3 expression in NKT cells**	7.06±4.07	5.46 (1.43-21.31)	7.57±5.39	5.75 (0.55-24.04)	1.61±1.51	0.88 (0.16-4.91)	1.23±0.73	1.07 (0.32-2.63)	0.944	0.000*	0.000*	0.000*	0.000*	0.983
**TLR-4 expression in NKT cells**	5.37±4.20	4.48 (0.92-17.39)	5.60±4.78	4.28 (0.11-20.50)	1.17±0.79	0.91 (0.23-2.40)	1.40±1.08	1.02 (0.36-4.66)	0.916	0.000*	0.000*	0.000*	0.000*	0.755
**TLR-9 expression in NKT cells**	8.63±4.77	7.54 (0.38-17.74)	7.93±5.60	6.57 (0.40-22.86)	1.03±0.51	0.89 (0.31-2.17)	1.33±0.93	1.07 (0.24-4.21)	0.377	0.000*	0.000*	0.000*	0.000*	0.573

Supplementary materials Tabel S5. The influence of age of GC patients on the percentage of occurrence and level of expression of TLR-2, -3, -4 and -9 on NK and NKT-like cells with particular emphasis on differences between the diffuse and intestinal type.

**Parameters**	**Diffuse ≤50**		**Intestinal ≤50**		**Diffuse ≥51**		**Intestinal ≥51**		**p-Value**					
	**Mean±SD**	**Median (Range)**	**Mean±SD**	**Median (Range)**	**Mean±SD**	**Median (Range)**	**Mean±SD**	**Median (Range)**	**I vs. II**	**I vs. III**	**i vs. IV**	**II vs. III**	**II vs. IV**	**III vs. IV**
**CD45+ [%]**	84.50±12.44	88.53 (54.71-99.38)	91.14±5.83	94.98 (71.31-99.83)	84.57±12.2 3	87.81 (53.20-98.49)	87.69±8.20	90.20 (69.59-97.60)	0.107	0.930	0.496	0.087	0.169	0.579
**NK cells [%]**	10.06±4.09	8.87 (3.69-17.84)	12.47±4.95	11.79 (7.14-25.19)	10.12±4.57	9.78 (3.05-18.51)	12.87±4.69	13.00 (4.23-20.62)	0.193	0.992	0.082	0.168	0.592	0.040*
**CD3-CD56+^{dim} [%]**	8.42±3.53	7.51	10.70±4.53	9.96	8.51±4.13	7.92	10.94±4.12	11.57	0.206	0.946	0.077	0.129	0.592	0.057

		(2.99-15.32)		(5.71-22.42)		(2.26-16.47)		(3.76-17.53)						
**CD3-CD56+^{bright}[%]**	1.39±0.69	1.29 (0.46-3.05)	1.52±0.61	1.38 (0.65-2.55)	1.37±0.65	1.32 (0.35-2.93)	1.69±0.78	1.60 (0.23-3.43)	0.536	0.884	0.217	0.283	0.569	0.097
**CD3+CD56+ [%]**	2.98±2.17	2.19 (0.61-6.85)	3.46±2.28	3.33 (0.27-7.22)	3.82±2.30	3.39 (0.84-8.95)	4.61±3.26	3.34 (0.53-11.94)	0.464	0.143	0.149	0.687	0.341	0.579
**CD3+ [%]**	66.96±10.13	69.50 (45.54-81.70)	70.84±7.25	73.29 (55.98-82.85)	66.92±11.10	67.45 (39.88-88.04)	66.54±8.34	66.00 (53.49-81.09)	0.283	0.977	0.806	0.243	0.180	0.682
**CD19 B lymphocytes [%]**	7.48±3.15	7.27 (1.66-13.71)	7.83±4.04	6.42 (2.91-16.48)	7.54±3.98	6.38 (1.88-20.90)	8.29±3.63	7.32 (2.96-15.23)	0.896	0.792	0.633	0.863	0.545	0.397
**CD3+CD4+ T lymphocytes [%]**	34.02±10.32	32.86 (19.68-57.40)	37.34±11.04	39.21 (14.60-52.85)	35.07±10.68	34.30 (16.25-59.61)	34.37±10.07	32.12 (14.28-57.21)	0.319	0.660	0.919	0.440	0.359	0.849
**CD3+CD8+ T lymphocytes [%]**	30.04±8.24	30.04 (8.24-	30.05±5.65	30.33 (17.97-40.08)	28.04±9.20	28.38 (9.00-43.91)	28.97±10.34	28.55 (11.79-48.70)	1.000	0.513	0.675	0.454	0.592	0.935
**CD3+CD4+/CD3+CD8+ cells ratio**	1.27±0.66	1.00 (0.43-2.95)	1.33±0.53	1.38 (0.36-2.09)	1.47±0.83	1.20 (0.54-3.70)	1.47±0.95	1.34 (0.38-4.23)	0.587	0.538	0.675	0.954	0.769	0.834
**CD3-CD56+^{dim}TLR-2+ [%]**	11.06±3.20	11.42 (4.04-14.99)	9.76±2.99	10.68 (4.78-13.30)	10.74±2.61	11.04 (4.76-14.78)	8.60±2.59	7.99 (4.97-13.73)	0.235	0.513	0.016*	0.283	0.341	0.006*
**CD3-CD56+^{dim}TLR-3+ [%]**	8.97±.03	9.40 (2.13-13.91)	8.69±2.21	9.30 (3.19-11.10)	9.04±3.02	9.08 (2.36-13.88)	7.30±.53	7.55 (2.02-13.67)	0.536	0.838	0.112	0.671	0.169	0.073
**CD3-CD56+^{dim}TLR-4+ [%]**	10.95±2.48	11.60 (4.87-13.91)	10.85±2.66	12.09 (5.27-13.41)	10.45±2.90	11.38 (4.25-13.96)	8.79±2.87	8.38 (4.39-13.99)	0.896	0.807	0.035*	0.722	0.043*	0.062
**CD3-CD56+^{dim}TLR-9+ [%]**	9.93±3.30	10.24 (2.52-14.24)	9.62±2.96	10.20 (3.29-13.68)	9.36±2.77	10.23 (2.02-14.32)	7.36±2.84	6.32 (2.44-12.22)	0.779	0.384	0.009*	0.774	0.017*	0.022*
**CD3-CD56+^{bright}TLR-2+ [%]**	9.15±3.10	9.66 (2.71-13.07)	8.04±2.80	8.12 (3.68-11.84)	8.64±2.74	8.35 (3.19-15.22)	6.58±2.55	5.63 (3.83-13.18)	0.220	0.373	0.016*	0.621	0.192	0.006*
**CD3-CD56+^{bright}TLR-3+ [%]**	7.45±2.81	7.94 (1.43-11.96)	7.21±2.33	8.28 (2.46-9.88)	7.38±2.99	7.88 (1.58-12.97)	5.69±3.22	5.81 (1.35-14.08)	0.955	0.946	0.055	0.739	0.112	0.060
**CD3-CD56+^{bright}TLR-4+ [%]**	9.04±2.65	9.72 (3.75-13.88)	8.95±2.64	9.80 (3.90-11.56)	8.47±2.98	9.45 (2.85-12.52)	6.75±2.68	5.86 (2.94-11.93)	0.750	0.632	0.035*	0.756	0.039*	0.033*
**CD3-CD56+^{bright}TLR-9+ [%]**	8.27±3.18	8.75 (1.69-14.67)	7.93±2.80	8.23 (2.53-11.76)	7.57±2.71	7.55 (1.35-12.82)	5.61±2.46	4.57 (1.88-12.12)	0.837	0.343	0.005*	0.654	0.014*	0.011*
**CD3+CD56+TLR-2+ [%]**	8.82±2.95	9.11 (3.00-12.91)	8.22±1.72	8.33 (5.67-12.15)	8.29±2.77	8.47 (3.20-12.97)	6.71±2.70	5.56 (3.02-12.53)	0.357	0.538	0.044*	0.881	0.051	0.057
**CD3+CD56+TLR-3+ [%]**	7.77±2.63	7.58 (3.21-12.26)	7.46±2.33	7.62 (3.08-13.30)	7.58±2.46	7.35 (3.77-13.72)	6.44±2.48	6.39 (3.07-12.40)	0.722	0.747	0.158	0.936	0.306	0.156
**CD3+CD56+TLR-4+ [%]**	8.58±2.52	8.51 (3.84-13.13)	7.90±1.84	8.46 (3.74-9.69)	8.16±2.37	8.34 (3.45-13.46)	6.94±2.17	6.37 (3.30-12.65)	0.488	0.551	0.030*	1.000	0.138	0.038*

**CD3+CD56+TLR-9+ [%]**	7.61±3.16	7.44 (2.09-12.10)	7.00±2.22	6.84 (2.35-11.91)	7.80±2.79	7.96 (2.68-12.91)	5.68±2.42	5.64 (2.01-9.61)	0.667	0.853	0.059	0.253	0.192	0.011*
**TLR-2 expression in NK cells**	19.41±11.63	20.01 (0.97-40.09)	8.76±4.51	9.02 (1.08-17.33)	15.36±10.98	11.12 (1.22-37.66)	7.60±6.59	6.00 (0.25-26.35)	0.009*	0.313	0.001*	0.141	0.396	0.008*
**TLR-3 expression in NK cells**	10.55±5.97	10.74 (0.75-20.55)	5.03±1.73	4.70 (2.70-9.23)	8.87±6.06	6.51 (2.15-22.62)	4.72±3.43	4.78 (0.58-15.54)	0.011*	0.363	0.002*	0.141	0.616	0.012*
**TLR-4 expression in NK cells**	18.18± 8.59	19.65 (1.51-32.01)	8.60±4.20	9.63 (1.85-16.58)	13.82±10.05	10.06 (2.15-45.11)	7.15±5.66	5.53 (0.34-18.65)	0.001*	0.127	0.000*	0.208	0.377	0.008*
**TLR-9 expression in NK cells**	17.02±9.44	18.18 (0.10-30.13)	8.71±5.02	8.01 (2.68-21.26)	14.55±10.98	11.15 (0.38-41.83)	7.21±5.59	5.55 (1.23-22.66)	0.014*	0.428	0.002*	0.168	0.274	0.013*
**TLR-2 expression in NKT cells**	9.97±10.70	3.96 (0.69-43.56)	4.35±2.68	3.60 (0.51-8.43)	7.42±6.91	4.07 (0.70-23.10)	4.24±4.85	1.94 (0.28-16.80)	0.193	0.564	0.017*	0.512	0.231	0.046*
**TLR-3 expression in NKT cells**	6.67±3.43	5.78 (1.43-11.75)	7.08±4.66	5.30 (2.53-21.31)	8.05±5.70	5.46 (1.97-24.04)	6.79±4.76	6.16 (0.55-18.79)	0.896	0.718	0.828	0.918	0.849	0.579
**TLR-4 expression in NKT cells**	4.99±3.94	3.73 (0.92-14.88)	5.45±4.47	4.60 (1.38-17.39)	6.36±5.19	4.33 (0.20-20.50)	4.36±3.71	3.40 (0.11-14.47)	0.722	0.488	0.613	0.845	0.457	0.218
**TLR-9 expression in NKT cells**	9.08±5.46	7.54 (0.38-17.74)	7.71±3.63	7.53 (2.69-15.30)	9.18±5.65	8.57 (0.82-22.86)	5.85±4.84	5.14 (0.40-19.56)	0.587	0.992	0.072	0.542	0.097	0.032*

Supplementary materials Tabel S6. Spearman Rank Correlations

Para zmiennych	R	t(N-2)	p-Value
**CD3+CD8+ T lymphocytes [%] & CD3+CD4+ / T CD3+CD8+ cells ratio**	-0.848	-14.687	0.000*
**Stage & CD56+^{dim} [%]**	-0.530	-5.735	0.000*
**Stage & NK cells [%]**	-0.504	-5.344	0.000*
**CD3-CD56+^{dim} [%] & TLR2 expression in NKT cells**	-0.483	-5.056	0.000*
**CD3-CD56+^{dim} [%] & CD3-CD56+^{dim} TLR3+ [%]**	-0.482	-5.036	0.000*
**CD3-CD56+^{dim} [%] & TLR9 expression in NKT cells**	-0.473	-4.919	0.000*
**CD3-CD56+^{dim} [%] & CD3-CD56+^{dim} TLR9+ [%]**	-0.467	-4.840	0.000*
**CD3-CD56+^{dim} [%] & TLR4 expression in NKT cells**	-0.466	-4.827	0.000*
**NK cells [%] & CD56+^{dim} TLR3+ [%]**	-0.457	-4.708	0.000*
**CD56+^{dim} [%] & CD3+CD56+TLR2+ [%]**	-0.455	-4.679	0.000*
**NK cells [%] & TLR9 expression in NKT cells**	-0.449	-4.602	0.000*
**CD56+^{dim} [%] & TLR2 expression in NK cells**	-0.449	-4.602	0.000*
**NK cells [%] & TLR2 expression in NKT cells**	-0.448	-4.587	0.000*
**CD56+^{dim} [%] & CD3+CD56+TLR4+ [%]**	-0.447	-4.580	0.000*
**NK cells [%] & TLR4 expression in NKT cells**	-0.445	-4.555	0.000*
**NK cells [%] & CD56+ y TLR9+ [%]**	-0.445	-4.555	0.000*
**CD56+^{dim} [%] & CD56+^{bright} TLR9+ [%]**	-0.442	-4.514	0.000*
**CD56+^{dim} [%] & CD56+^{bright} TLR3+ [%]**	-0.439	-4.482	0.000*
**CD56+^{dim} [%] & TLR9 expression in NK cells**	-0.434	-4.412	0.000*

**CD56+^{dim} [%] & TLR3 expression in NK cells**	-0.429	-4.359	0.000*
**CD3+CD4+ T lymphocytes [%] & CD3+CD8+ T lymphocytes [%]**	-0.428	-4.341	0.000*
**CD3+CD8+ T lymphocytes [%] & CD3+CD4+ T lymphocytes [%]**	-0.428	-4.341	0.000*
**NK cells [%] & CD3+CD56+TLR2+ [%]**	-0.426	-4.318	0.000*
**NK cells [%] & TLR2 expression in NK cells**	-0.424	-4.287	0.000*
**NK cells [%] & CD3+CD56+TLR4+ [%]**	-0.421	-4.252	0.000*
**CD3+CD56+ [%] & CD3+CD56+TLR2+ [%]**	-0.419	-4.234	0.000*
**NK cells [%] & CD3-CD56+^{bright} TLR9+ [%]**	-0.416	-4.191	0.000*
**NK cells [%] & CD3-CD56+^{bright} TLR3+ [%]**	-0.413	-4.158	0.000*
**NK cells [%] & TLR9 expression in NK cells**	-0.412	-4.138	0.000*
**NK cells [%] & TLR3 expression in NK cells**	-0.408	-4.101	0.000*
**CD56+^{dim} [%] & CD3+CD56+TLR9+ [%]**	-0.396	-3.955	0.000*
**CD56+^{dim} [%] & TLR3 expression in NKT cells**	-0.383	-3.802	0.000*
**CD56+^{dim} [%] & CD3-CD56+^{bright} TLR2+ [%]**	-0.373	-3.682	0.000*
**CD56+^{dim} [%] & TLR4 expression in NK cells**	-0.373	-3.679	0.000*
**CD56+^{dim} [%] & CD3-CD56+^{dim} TLR2+ [%]**	-0.369	-3.640	0.000*
**NK cells [%] & TLR3 expression in NKT cells**	-0.369	-3.634	0.000*
**CD3+CD56+ [%] & CD3+CD4+ T lymphocytes [%]**	-0.368	-3.633	0.000*
**NK cells [%] & CD3+CD56+TLR9+ [%]**	-0.368	-3.632	0.000*
**CD3+CD56+ [%] & TLR3 expression in NKT cells**	-0.360	-3.537	0.001*
**Stage & CD3+CD56+ [%]**	-0.357	-3.499	0.001*
**NK cells [%] & CD3-CD56+^{dim} TLR2+ [%]**	-0.356	-3.490	0.001*
**NK cells [%] & CD3-CD56+^{bright} TLR2+ [%]**	-0.351	-3.439	0.001*
**CD56+^{dim} [%] & CD3-CD56+^{bright} TLR4+ [%]**	-0.347	-3.394	0.001*
**NK cells [%] & TLR4 expression in NK cells**	-0.347	-3.388	0.001*
**CD56+^{dim} [%] & CD3+CD56+TLR3+ [%]**	-0.344	-3.358	0.001*
**CD19+ B lymphocytes [%] & CD56+ dim TLR4+ [%]**	-0.339	-3.303	0.001*
**CD3+CD56+ [%] & CD3+CD56+TLR3+ [%]**	-0.331	-3.219	0.002*
**CD19+ B lymphocytes [%] & CD3+CD4+ T lymphocytes [%]**	-0.331	-3.210	0.002*
**CD19+ B lymphocytes [%] & CD3+CD56+TLR2+ [%]**	-0.325	-3.150	0.002*
**CD3+CD56+ [%] & CD56+^{dim} TLR2+ [%]**	-0.325	-3.145	0.002*
**NK cells [%] & CD56+^{bright} TLR4+ [%]**	-0.321	-3.111	0.003*
**NK cells [%] & CD3+CD56+TLR3+ [%]**	-0.321	-3.107	0.003*
**CD3+CD56+ [%] & CD56+^{dim} TLR4+ [%]**	-0.320	-3.095	0.003*
**CD3+CD56+ [%] & CD56+^{dim} TLR3+ [%]**	-0.319	-3.090	0.003*
**CD56+^{dim} [%] & CD56+^{dim} TLR4+ [%]**	-0.313	-3.020	0.003*
**CD3+CD56+ [%] & CD56+^{dim} TLR9+ [%]**	-0.312	-3.006	0.003*
**CD3+CD56+ [%] & TLR2 expression in NK cells**	-0.301	-2.893	0.005*
**CD3+ [%] & CD19+ B lymphocytes [%]**	-0.300	-2.885	0.005*
**CD3+CD56+ [%] & CD56+^{bright} TLR3+ [%]**	-0.298	-2.859	0.005*
**NK cells [%] & CD56+^{dim} TLR4+ [%]**	-0.298	-2.857	0.005*
**CD19+ B lymphocytes [%] & TLR2 expression in NKT cells**	-0.297	-2.847	0.006*
**CD3+CD56+ [%] & CD56+^{bright} TLR4+ [%]**	-0.294	-2.816	0.006*
**CD3+CD56+ [%] & TLR4 expression in NKT cells**	-0.287	-2.744	0.007*
**CD3+CD56+ [%] & TLR9 expression in NK cells**	-0.284	-2.715	0.008*
**CD3+CD56+ [%] & CD56+^{bright} TLR2+ [%]**	-0.282	-2.697	0.008*
**Type of GC & CD56+^{dim} TLR2+ [%]**	-0.280	-2.672	0.009*
**CD3+CD56+ [%] & CD3+CD56+TLR4+ [%]**	-0.279	-2.658	0.009*
**CD3+CD56+ [%] & TLR9 expression in NKT cells**	-0.269	-2.559	0.012*
**CD3+CD56+ [%] & CD56+^{bright} TLR9+ [%]**	-0.269	-2.558	0.012*
**Type of GC & CD56+^{bright} TLR2+ [%]**	-0.268	-2.554	0.012*
**CD3+CD56+ [%] & TLR4 expression in NK cells**	-0.266	-2.527	0.013*
**CD19+ B lymphocytes [%] & CD3+CD56+TLR9+ [%]**	-0.263	-2.494	0.015*
**CD3+CD56+ [%] & TLR3 expression in NK cells**	-0.262	-2.484	0.015*
**CD3+CD56+ [%] & TLR2 expression in NKT cells**	-0.259	-2.455	0.016*
**Age & CD56+^{bright} TLR9+ [%]**	-0.254	-2.406	0.018*
**CD19+ B lymphocytes [%] & CD3+CD4+ / T CD3+CD8+ cells ratio**	-0.253	-2.399	0.019*
**CD19+ B lymphocytes [%] & CD56+^{dim} TLR9+ [%]**	-0.253	-2.399	0.019*

**CD19+ B lymphocytes [%] & TLR4 expression in NKT cells**	-0.250	-2.371	0.020*
**Stage & CD56+bright [%]**	-0.247	-2.338	0.022*
**CD3+CD56+ [%] & CD3+CD56+TLR9+ [%]**	-0.247	-2.332	0.022*
**Stage & Type of GC**	-0.242	-2.283	0.025*
**CD3+CD56+ [%] & CD3+CD4+ / T CD3+CD8+ cells ratio**	-0.241	-2.273	0.026*
**CD19+ B lymphocytes [%] & TLR2 expression in NK cells**	-0.238	-2.251	0.027*
**CD19+ B lymphocytes [%] & TLR9 expression in NK cells**	-0.238	-2.241	0.028*
**CD56+bright [%] & CD3-CD56+ ^{dim} TLR9+ [%]**	-0.235	-2.213	0.030*
**Type of GC & CD3+CD56+TLR9+ [%]**	-0.234	-2.207	0.030*
**Stage & CD19+ B lymphocytes [%]**	-0.231	-2.173	0.033*
**Age & CD3-CD56+ ^{dim} TLR9+ [%]**	-0.229	-2.159	0.034*
**CD19+ B lymphocytes [%] & TLR4 expression in NK cells**	-0.228	-2.149	0.034*
**CD56+^{bright} [%] & TLR3 expression in NKT cells**	-0.222	-2.086	0.040*
**CD56+^{bright} [%] & CD3-CD56+ ^{dim} TLR3+ [%]**	-0.221	-2.073	0.041*
**CD19+ B lymphocytes [%] & TLR3 expression in NKT cells**	-0.218	-2.046	0.044*
**CD19+ B lymphocytes [%] & TLR3 expression in NK cells**	-0.217	-2.034	0.045*
**Gender & CD3+CD56+TLR2+ [%]**	0.214	2.006	0.048*
**Gender & TLR9 expression in NKT cells**	0.222	2.088	0.040*
**Gender & CD3+CD56+TLR4+ [%]**	0.226	2.122	0.037*
**Gender & CD3-CD56+ ^{dim} TLR3+ [%]**	0.226	2.127	0.036*
**Gender & TLR4 expression in NK cells**	0.230	2.166	0.033*
**CD3+CD8+ T lymphocytes [%] & TLR3 expression in NKT cells**	0.236	2.226	0.029*
**Type of GC & CD3-CD56+ ^{dim} [%]**	0.245	2.312	0.023*
**Type of GC & NK cells [%]**	0.247	2.336	0.022*
**CD3+ [%] & CD56+ ^{dim} TLR3+ [%]**	0.249	2.356	0.021*
**CD3+ [%] & CD3+CD56+TLR2+ [%]**	0.254	2.408	0.018*
**Gender & CD3-CD56+ ^{dim} TLR4+ [%]**	0.255	2.417	0.018*
**CD3+ [%] & TLR9 expression in NKT cells**	0.270	2.573	0.012*
**CD3+ [%] & CD3-CD56+ ^{dim} TLR2+ [%]**	0.276	2.634	0.010*
**CD3+ [%] & TLR3 expression in NKT cells**	0.281	2.688	0.009*
**CD3+ [%] & CD3+CD56+TLR4+ [%]**	0.287	2.744	0.007*
**Stage & CD3+ [%]**	0.292	2.800	0.006*
**CD3+ [%] & CD56+ ^{dim} TLR9+ [%]**	0.304	2.928	0.004*
**CD3+ [%] & CD3+CD56+TLR9+ [%]**	0.305	2.934	0.004*
**CD3+CD56+TLR3+ [%] & TLR3 expression in NKT cells**	0.319	3.082	0.003*
**CD56+^{bright} TLR2+ [%] & TLR3 expression in NKT cells**	0.327	3.173	0.002*
**CD56+^{bright} [%] & CD3+CD56+ [%]**	0.328	3.187	0.002*
**CD56+^{bright} TLR9+ [%] & TLR3 expression in NKT cells**	0.332	3.225	0.002*
**CD3+ [%] & CD3+CD56+TLR3+ [%]**	0.333	3.238	0.002*
**CD56+^{bright} TLR3+ [%] & TLR3 expression in NKT cells**	0.336	3.271	0.002*
**CD45+ [%] & CD3+CD4+ T lymphocytes [%]**	0.337	3.278	0.002*
**CD3+ [%] & CD3+CD8+ T lymphocytes [%]**	0.338	3.289	0.001*
**CD56+^{bright} TLR4+ [%] & TLR3 expression in NKT cells**	0.344	3.353	0.001*
**CD45+ [%] & CD3+CD8+ T lymphocytes [%]**	0.357	3.505	0.001*
**CD56+ ^{dim} [%] & CD3+CD56+ [%]**	0.364	3.583	0.001*
**NK cells [%] & CD3+CD56+ [%]**	0.373	3.682	0.000*
**CD3+ [%] & TLR4 expression in NKT cells**	0.375	3.709	0.000*
**TLR4 expression in NK cells & TLR3 expression in NKT cells**	0.378	3.745	0.000*
**CD56+^{bright} TLR4+ [%] & TLR4 expression in NKT cells**	0.388	3.864	0.000*
**CD3+CD56+TLR3+ [%] & TLR4 expression in NKT cells**	0.391	3.890	0.000*
**TLR3 expression in NK cells & TLR3 expression in NKT cells**	0.398	3.982	0.000*
**CD56+ ^{dim} TLR4+ [%] & TLR4 expression in NKT cells**	0.401	4.013	0.000*
**CD56+ ^{dim} TLR3+ [%] & TLR3 expression in NKT cells**	0.405	4.058	0.000*
**CD56+ ^{dim} TLR4+ [%] & TLR3 expression in NKT cells**	0.417	4.201	0.000*
**TLR9 expression in NK cells & TLR3 expression in NKT cells**	0.427	4.327	0.000*
**CD56+ ^{dim} TLR9+ [%] & TLR3 expression in NKT cells**	0.432	4.394	0.000*
**TLR2 expression in NK cells & TLR3 expression in NKT cells**	0.445	4.548	0.000*
**CD56+ ^{dim} TLR2+ [%] & TLR3 expression in NKT cells**	0.448	4.591	0.000*

CD45+ [%] & CD3-CD56+bright [%]	0.451	4.631	0.000*	CD56+ dim TLR4+ [%] & TLR9 expression in NKT cells	0.611	7.075	0.000*
CD3+CD56+TLR2+ [%] & TLR3 expression in NKT cells	0.458	4.725	0.000*	CD56+bright TLR2+ [%] & CD3+CD56+TLR3+ [%]	0.615	7.140	0.000*
CD45+ [%] & CD3-CD56+ dim [%]	0.461	4.761	0.000*	CD3+CD56+TLR3+ [%] & TLR2 expression in NKT cells	0.616	7.159	0.000*
CD3+CD56+TLR9+ [%] & TLR3 expression in NKT cells	0.465	4.815	0.000*	TLR9 expression in NK cells & TLR9 expression in NKT cells	0.619	7.217	0.000*
TLR4 expression in NK cells & TLR4 expression in NKT cells	0.468	4.858	0.000*	CD3+CD56+TLR2+ [%] & TLR4 expression in NKT cells	0.626	7.351	0.000*
CD45+ [%] & NK cells [%]	0.472	4.901	0.000*	CD56+bright TLR4+ [%] & TLR9 expression in NKT cells	0.628	7.400	0.000*
CD3-CD56+bright TLR3+ [%] & TLR4 expression in NKT cells	0.477	4.969	0.000*	CD3+CD56+TLR3+ [%] & TLR3 expression in NK cells	0.633	7.495	0.000*
CD3+CD56+TLR4+ [%] & TLR3 expression in NKT cells	0.484	5.067	0.000*	CD3+CD56+TLR3+ [%] & TLR9 expression in NK cells	0.634	7.508	0.000*
CD3-CD56+bright TLR2+ [%] & TLR4 expression in NKT cells	0.489	5.144	0.000*	CD3+CD56+TLR2+ [%] & TLR9 expression in NKT cells	0.637	7.583	0.000*
Stage & TLR3 expression in NKT cells	0.497	5.253	0.000*	CD56+ dim TLR4+ [%] & CD3+CD56+TLR3+ [%]	0.639	7.623	0.000*
CD3-CD56+bright TLR9+ [%] & TLR4 expression in NKT cells	0.498	5.270	0.000*	CD56+ dim TLR2+ [%] & CD3+CD56+TLR3+ [%]	0.642	7.668	0.000*
TLR9 expression in NK cells & TLR4 expression in NKT cells	0.518	5.551	0.000*	CD3+CD56+TLR3+ [%] & CD3+CD56+TLR4+ [%]	0.651	7.854	0.000*
TLR3 expression in NK cells & TLR4 expression in NKT cells	0.525	5.649	0.000*	CD56+ dim TLR9+ [%] & CD3+CD56+TLR3+ [%]	0.652	7.879	0.000*
CD3+CD56+TLR3+ [%] & TLR9 expression in NKT cells	0.525	5.653	0.000*	CD56+ dim TLR2+ [%] & CD56+bright TLR4+ [%]	0.655	7.936	0.000*
CD3-CD56+ dim TLR3+ [%] & TLR4 expression in NKT cells	0.539	5.860	0.000*	CD56+ dim TLR9+ [%] & TLR9 expression in NKT cells	0.660	8.059	0.000*
TLR2 expression in NK cells & TLR4 expression in NKT cells	0.544	5.939	0.000*	CD56+bright TLR3+ [%] & TLR9 expression in NKT cells	0.661	8.077	0.000*
CD3+CD56+TLR9+ [%] & TLR4 expression in NKT cells	0.544	5.947	0.000*	CD56+ dim TLR2+ [%] & TLR9 expression in NKT cells	0.664	8.134	0.000*
CD3-CD56+ dim TLR2+ [%] & TLR4 expression in NKT cells	0.546	5.968	0.000*	TLR3 expression in NKT cells & TLR9 expression in NKT cells	0.665	8.165	0.000*
CD3-CD56+ dim TLR9+ [%] & TLR4 expression in NKT cells	0.556	6.132	0.000*	CD56+bright TLR2+ [%] & TLR9 expression in NKT cells	0.667	8.200	0.000*
CD3+CD56+TLR3+ [%] & TLR4 expression in NK cells	0.565	6.283	0.000*	TLR2 expression in NK cells & TLR9 expression in NKT cells	0.668	8.218	0.000*
CD3-CD56+bright TLR4+ [%] & CD3+CD56+TLR3+ [%]	0.579	6.504	0.000*	CD56+bright TLR3+ [%] & CD3+CD56+TLR3+ [%]	0.668	8.227	0.000*
TLR4 expression in NKT cells & TLR9 expression in NKT cells	0.594	6.769	0.000*	CD56+bright TLR9+ [%] & TLR9 expression in NKT cells	0.669	8.244	0.000*
CD3+CD56+TLR4+ [%] & TLR4 expression in NKT cells	0.595	6.790	0.000*	CD56+ dim TLR9+ [%] & CD56+bright TLR4+ [%]	0.669	8.246	0.000*
TLR4 expression in NK cells & TLR9 expression in NKT cells	0.600	6.874	0.000*	CD56+ dim TLR9+ [%] & TLR2 expression in NKT cells	0.673	8.335	0.000*
TLR3 expression in NK cells & TLR9 expression in NKT cells	0.600	6.882	0.000*	CD56+bright TLR4+ [%] & CD3+CD56+TLR9+ [%]	0.674	8.360	0.000*
CD3+ [%] & CD3+CD4+ T lymphocytes [%]	0.601	6.899	0.000*	CD3+CD56+TLR3+ [%] & TLR2 expression in NK cells	0.674	8.365	0.000*
Stage & TLR4 expression in NKT cells	0.608	7.011	0.000*	CD56+ dim TLR9+ [%] & TLR4 expression in NK cells	0.675	8.395	0.000*
CD3+CD56+TLR3+ [%] & CD56+bright TLR9+ [%]	0.608	7.026	0.000*	CD56+bright TLR4+ [%] & CD3+CD56+TLR2+ [%]	0.677	8.429	0.000*
TLR2 expression in NKT cells & TLR3 expression in NKT cells	0.609	7.045	0.000*	CD56+bright TLR4+ [%] & TLR3 expression in NK cells	0.683	8.580	0.000*

**CD3-CD56+^{bright} TLR4+ [%] & CD3+CD56+TLR4+ [%]**	0.684	8.600	0.000*
**CD3-CD56+ ^{dim} TLR2+ [%] & CD56+ ^{dim} TLR4+ [%]**	0.684	8.602	0.000*
**TLR2 expression in NKT cells & TLR4 expression in NKT cells**	0.688	8.686	0.000*
**CD3-CD56+ ^{dim} TLR4+ [%] & TLR3 expression in NK cells**	0.688	8.692	0.000*
**CD3-CD56+ ^{dim} TLR3+ [%] & TLR9 expression in NKT cells**	0.690	8.739	0.000*
**CD3-CD56+ ^{dim} TLR2+ [%] & CD56+bright TLR3+ [%]**	0.692	8.782	0.000*
**CD3-CD56+^{bright} TLR4+ [%] & TLR9 expression in NK cells**	0.693	8.799	0.000*
**CD3-CD56+ ^{dim} TLR9+ [%] & TLR3 expression in NK cells**	0.700	8.974	0.000*
**CD3-CD56+ ^{dim} TLR9+ [%] & CD56+^{bright} TLR3+ [%]**	0.703	9.056	0.000*
**CD3-CD56+ ^{dim} TLR2+ [%] & TLR2 expression in NKT cells**	0.708	9.178	0.000*
**CD3-CD56+ ^{dim} TLR4+ [%] & CD56+ ^{dim} TLR9+ [%]**	0.711	9.259	0.000*
**CD3-CD56+ ^{dim} TLR4+ [%] & TLR9 expression in NK cells**	0.712	9.284	0.000*
**CD3-CD56+ ^{dim} TLR4+ [%] & CD3+CD56+TLR9+ [%]**	0.713	9.308	0.000*
**CD3-CD56+ ^{dim} TLR2+ [%] & CD56+^{bright} TLR9+ [%]**	0.714	9.355	0.000*
**CD3+CD56+TLR3+ [%] & CD3+CD56+TLR9+ [%]**	0.715	9.367	0.000*
**TLR3 expression in NKT cells & TLR4 expression in NKT cells**	0.717	9.415	0.000*
**CD3-CD56+ ^{dim} TLR4+ [%] & TLR2 expression in NKT cells**	0.717	9.430	0.000*
**CD3-CD56+ ^{dim} TLR4+ [%] & CD56+^{bright} TLR2+ [%]**	0.718	9.444	0.000*
**CD45+ [%] & CD3+ [%]**	0.718	9.447	0.000*
**CD3-CD56+^{bright} TLR9+ [%] & TLR2 expression in NKT cells**	0.718	9.468	0.000*
**CD3-CD56+ ^{dim} TLR4+ [%] & CD3+CD56+TLR2+ [%]**	0.719	9.469	0.000*
**CD3+CD56+TLR2+ [%] & CD3+CD56+TLR3+ [%]**	0.719	9.474	0.000*
**CD3-CD56+^{bright} TLR4+ [%] & TLR2 expression in NKT cells**	0.721	9.523	0.000*
**CD3-CD56+ ^{dim} TLR4+ [%] & CD56+^{bright} TLR9+ [%]**	0.722	9.572	0.000*
**CD3-CD56+ ^{dim} [%] & CD56+^{bright} [%]**	0.723	9.581	0.000*
**CD3+CD56+TLR4+ [%] & TLR9 expression in NKT cells**	0.724	9.610	0.000*
**CD3-CD56+ ^{dim} TLR4+ [%] & TLR4 expression in NK cells**	0.725	9.636	0.000*
**CD3-CD56+ ^{dim} TLR9+ [%] & TLR9 expression in NK cells**	0.726	9.663	0.000*
**CD3-CD56+ ^{dim} TLR2+ [%] & TLR4 expression in NK cells**	0.726	9.680	0.000*
**CD56+^{bright} TLR4+ [%] & TLR4 expression in NK cells**	0.727	9.700	0.000*
**CD56+ ^{dim} TLR9+ [%] & CD56+^{bright} TLR2+ [%]**	0.729	9.752	0.000*
**CD56+^{bright} TLR9+ [%] & CD3+CD56+TLR9+ [%]**	0.730	9.778	0.000*
**CD56+ ^{dim} TLR9+ [%] & CD3+CD56+TLR9+ [%]**	0.730	9.779	0.000*
**CD56+ ^{dim} TLR2+ [%] & TLR3 expression in NK cells**	0.730	9.801	0.000*
**TLR4 expression in NK cells & TLR2 expression in NKT cells**	0.731	9.822	0.000*
**CD56+ ^{dim} TLR2+ [%] & CD56+ ^{dim} TLR9+ [%]**	0.733	9.890	0.000*
**CD56+^{bright} TLR2+ [%] & CD3+CD56+TLR2+ [%]**	0.734	9.914	0.000*
**CD56+^{bright} TLR9+ [%] & CD3+CD56+TLR2+ [%]**	0.734	9.917	0.000*
**CD56+^{bright} TLR9+ [%] & TLR4 expression in NK cells**	0.734	9.919	0.000*
**CD56+ ^{dim} TLR2+ [%] & CD3+CD56+TLR2+ [%]**	0.735	9.941	0.000*
**CD56+^{bright} TLR9+ [%] & TLR3 expression in NK cells**	0.735	9.948	0.000*
**CD56+^{bright} TLR2+ [%] & TLR2 expression in NKT cells**	0.736	9.959	0.000*
**CD56+ ^{dim} TLR4+ [%] & CD3+CD56+TLR4+ [%]**	0.736	9.965	0.000*
**CD56+^{bright} TLR4+ [%] & TLR2 expression in NK cells**	0.737	9.995	0.000*
**CD56+ ^{dim} TLR3+ [%] & CD3+CD56+TLR3+ [%]**	0.739	10.046	0.000*
**CD56+^{bright} TLR9+ [%] & TLR9 expression in NK cells**	0.740	10.098	0.000*
**TLR2 expression in NKT cells & TLR9 expression in NKT cells**	0.742	10.134	0.000*
**CD56+^{bright} TLR3+ [%] & TLR9 expression in NK cells**	0.743	10.177	0.000*
**CD56+^{bright} TLR3+ [%] & CD3+CD56+TLR4+ [%]**	0.744	10.205	0.000*
**CD56+ ^{dim} TLR4+ [%] & TLR2 expression in NK cells**	0.745	10.227	0.000*
**CD3+CD56+TLR9+ [%] & TLR4 expression in NK cells**	0.745	10.247	0.000*
**CD56+ ^{dim} TLR2+ [%] & TLR9 expression in NK cells**	0.749	10.359	0.000*
**CD56+^{bright} TLR3+ [%] & TLR4 expression in NK cells**	0.750	10.382	0.000*
**CD56+ ^{dim} TLR9+ [%] & TLR2 expression in NK cells**	0.752	10.440	0.000*
**CD56+^{bright} TLR3+ [%] & CD3+CD56+TLR9+ [%]**	0.752	10.460	0.000*
**CD56+^{bright} TLR3+ [%] & TLR2 expression in NKT cells**	0.753	10.473	0.000*
**CD56+^{bright} TLR2+ [%] & CD3+CD56+TLR4+ [%]**	0.753	10.480	0.000*
**CD3+CD56+TLR4+ [%] & TLR4 expression in NK cells**	0.753	10.497	0.000*

Stage & TLR9 expression in NKT cells	0.754	10.514	0.000*
TLR9 expression in NKT cells & CD3+CD56+TLR9+ [%]	0.755	10.538	0.000*
CD3-CD56+^{dim} TLR9+ [%] & CD3+CD56+TLR2+ [%]	0.755	10.547	0.000*
CD3-CD56+^{dim} TLR4+ [%] & CD56+^{bright} TLR3+ [%]	0.755	10.566	0.000*
CD3-CD56+^{bright} TLR3+ [%] & TLR3 expression in NK cells	0.759	10.675	0.000*
CD3-CD56+^{dim} TLR2+ [%] & CD56+^{dim} TLR3+ [%]	0.759	10.696	0.000*
CD3-CD56+^{bright} TLR3+ [%] & CD3+CD56+TLR2+ [%]	0.760	10.724	0.000*
CD3+CD56+TLR2+ [%] & TLR4 expression in NK cells	0.763	10.810	0.000*
CD56+^{bright} TLR2+ [%] & CD3+CD56+TLR9+ [%]	0.763	10.813	0.000*
CD3+CD56+TLR4+ [%] & TLR2 expression in NKT cells	0.764	10.868	0.000*
TLR3 expression in NK cells & TLR2 expression in NKT cells	0.765	10.873	0.000*
CD3-CD56+^{bright} TLR9+ [%] & CD3+CD56+TLR4+ [%]	0.765	10.877	0.000*
Stage & CD56+^{bright} TLR4+ [%]	0.765	10.893	0.000*
TLR2 expression in NKT cells & TLR9 expression in NK cells	0.767	10.972	0.000*
CD3-CD56+^{dim} TLR9+ [%] & CD56+^{dim} TLR3+ [%]	0.767	10.973	0.000*
CD3-CD56+^{bright} TLR2+ [%] & TLR9 expression in NK cells	0.772	11.114	0.000*
CD3-CD56+^{bright} TLR2+ [%] & TLR3 expression in NK cells	0.772	11.129	0.000*
CD3-CD56+^{dim} TLR2+ [%] & CD3+CD56+TLR9+ [%]	0.776	11.267	0.000*
CD3+CD56+TLR9+ [%] & TLR9 expression in NK cells	0.778	11.342	0.000*
CD3-CD56+^{dim} TLR2+ [%] & CD3+CD56+TLR4+ [%]	0.780	11.408	0.000*
CD3+CD56+TLR2+ [%] & TLR2 expression in NKT cells	0.780	11.412	0.000*
CD3+CD56+TLR2+ [%] & CD3+CD56+TLR4+ [%]	0.780	11.427	0.000*
NK cells [%] & CD56+^{bright} [%]	0.782	11.484	0.000*
CD3-CD56+^{dim} TLR3+ [%] & TLR2 expression in NKT cells	0.782	11.490	0.000*
CD3-CD56+^{dim} TLR3+ [%] & TLR4 expression in NK cells	0.782	11.492	0.000*
Stage & TLR4 expression in NK cells	0.784	11.565	0.000*
TLR2 expression in NK cells & CD56+^{dim} TLR2+ [%]	0.784	11.572	0.000*
TLR2 expression in NK cells & CD56+^{bright} TLR9+ [%]	0.784	11.585	0.000*
CD56+^{bright} TLR2+ [%] & TLR4 expression in NK cells	0.785	11.609	0.000*
CD3+CD56+TLR9+ [%] & TLR2 expression in NKT cells	0.788	11.726	0.000*
Stage & CD3+CD56+TLR3+ [%]	0.789	11.766	0.000*
CD3+CD56+TLR4+ [%] & TLR3 expression in NK cells	0.789	11.769	0.000*
CD56+^{dim} TLR3+ [%] & CD56+^{bright} TLR4+ [%]	0.790	11.821	0.000*
CD3+CD56+TLR4+ [%] & CD3+CD56+TLR9+ [%]	0.791	11.856	0.000*
CD3+CD56+TLR9+ [%] & TLR3 expression in NK cells	0.792	11.872	0.000*
CD56+^{dim} TLR3+ [%] & CD56+^{dim} TLR4+ [%]	0.796	12.042	0.000*
CD3+CD56+TLR4+ [%] & TLR9 expression in NK cells	0.796	12.058	0.000*
Stage & CD56+^{dim} TLR4+ [%]	0.798	12.115	0.000*
CD3+CD56+TLR2+ [%] & TLR3 expression in NK cells	0.798	12.118	0.000*
CD56+^{bright} TLR3+ [%] & TLR2 expression in NK cells	0.802	12.290	0.000*
CD56+^{dim} TLR3+ [%] & CD56+^{bright} TLR9+ [%]	0.802	12.306	0.000*
CD3+CD4+ T lymphocytes [%] & CD3+CD4+ / T CD3+CD8+ cells ratio	0.803	12.344	0.000*
CD56+^{dim} TLR3+ [%] & TLR9 expression in NK cells	0.805	12.443	0.000*
CD56+^{dim} TLR3+ [%] & TLR3 expression in NK cells	0.810	12.642	0.000*
CD56+^{dim} TLR3+ [%] & CD56+^{bright} TLR2+ [%]	0.810	12.644	0.000*
TLR2 expression in NK cells & TLR2 expression in NKT cells	0.811	12.722	0.000*
CD56+^{dim} TLR9+ [%] & CD3+CD56+TLR4+ [%]	0.815	12.910	0.000*
CD56+^{dim} TLR3+ [%] & CD3+CD56+TLR9+ [%]	0.817	12.985	0.000*
CD56+^{bright} TLR2+ [%] & TLR2 expression in NK cells	0.817	13.000	0.000*
CD3+CD56+TLR2+ [%] & CD3+CD56+TLR9+ [%]	0.825	13.361	0.000*
Stage & CD56+^{bright} TLR9+ [%]	0.827	13.461	0.000*
CD3+CD56+TLR4+ [%] & TLR2 expression in NK cells	0.828	13.524	0.000*
CD3+CD56+TLR2+ [%] & TLR9 expression in NK cells	0.829	13.611	0.000*
CD56+^{dim} TLR3+ [%] & CD3+CD56+TLR4+ [%]	0.836	13.957	0.000*
Stage & CD56+^{bright} TLR2+ [%]	0.837	14.008	0.000*
CD56+^{dim} TLR3+ [%] & CD3+CD56+TLR2+ [%]	0.840	14.174	0.000*
CD3+CD56+TLR9+ [%] & TLR2 expression in NK cells	0.841	14.252	0.000*
Stage & CD56+^{dim} TLR9+ [%]	0.841	14.266	0.000*

**Stage & TLR3 expression in NK cells**	0.843	14.354	0.000*
**CD3+CD56+TLR2+ [%] & TLR2 expression in NK cells**	0.843	14.376	0.000*
**Stage & CD56+ ^{dim} TLR2+ [%]**	0.844	14.434	0.000*
**CD56+^{bright} TLR2+ [%] & CD56+^{bright} TLR4+ [%]**	0.845	14.509	0.000*
**Stage & CD56+^{bright} TLR3+ [%]**	0.846	14.534	0.000*
**CD3-CD56+^{bright} TLR4+ [%] & CD56+^{bright} TLR9+ [%]**	0.849	14.757	0.000*
**CD3-CD56+ ^{dim} TLR3+ [%] & TLR2 expression in NK cells**	0.852	14.896	0.000*
**CD3-CD56+^{bright} TLR3+ [%] & CD56+^{bright} TLR9+ [%]**	0.853	14.988	0.000*
**CD3-CD56+^{bright} TLR2+ [%] & CD56+^{bright} TLR3+ [%]**	0.856	15.179	0.000*
**Stage & TLR9 expression in NK cells**	0.859	15.368	0.000*
**Stage & TLR2 expression in NKT cells**	0.861	15.522	0.000*
**CD3-CD56+^{bright} TLR2+ [%] & CD56+^{bright} TLR9+ [%]**	0.864	15.703	0.000*
**CD3-CD56+ ^{dim} TLR4+ [%] & CD56+^{bright} TLR4+ [%]**	0.877	16.743	0.000*
**CD3-CD56+^{bright} TLR3+ [%] & CD56+^{bright} TLR4+ [%]**	0.879	16.901	0.000*
**Stage & CD3+CD56+TLR4+ [%]**	0.882	17.153	0.000*
**Stage & TLR2 expression in NK cells**	0.899	18.845	0.000*
**Stage & CD3+CD56+TLR9+ [%]**	0.900	18.904	0.000*
**CD3-CD56+ ^{dim} TLR2+ [%] & CD56+^{bright} TLR2+ [%]**	0.903	19.231	0.000*
**Stage & CD3+CD56+TLR2+ [%]**	0.905	19.523	0.000*
**CD3-CD56+ ^{dim} TLR9+ [%] & CD56+bright TLR9+ [%]**	0.906	19.656	0.000*
**Stage & CD56+ ^{dim} TLR3+ [%]**	0.917	21.064	0.000*
**TLR4 expression in NK cells & TLR9 expression in NK cells**	0.918	21.268	0.000*
**TLR3 expression in NK cells & TLR4 expression in NK cells**	0.927	22.688	0.000*
**CD3-CD56+ ^{dim} TLR3+ [%] & CD56+^{bright} TLR3+ [%]**	0.944	26.308	0.000*
**TLR2 expression in NK cells & TLR4 expression in NK cells**	0.946	26.715	0.000*
**TLR3 expression in NK cells & TLR9 expression in NK cells**	0.957	30.320	0.000*
**TLR2 expression in NK cells & TLR9 expression in NK cells**	0.961	31.641	0.000*
**TLR2 expression in NK cells & TLR3 expression in NK cells**	0.975	39.856	0.000*
**NK cells [%] & CD56+ ^{dim} [%]**	0.994	81.217	0.000*

Supplementary materials Tabel S7. Tabulated summary of ROC curves for disease stage among GC patients.

	**CD56+^{dim}TLR-2+[%]**					
	I vs. II	I vs. III	I vs. IV	II vs. III	II vs. IV	III vs. IV
**Area**	0.74	1	1	0.9704	1	0.7979
**Std. Error**	0.09346	0	0	0.02242	0	0.05794
**95% confidence interval**	0,5568 to 0,9232	1,000 to 1,000	1,000 to 1,000	0,9264 to 1,000	1,000 to 1,000	0,6844 to 0,9115
**P value**	0.0251	<0,0001	<0,0001	<0,0001	<0,0001	0.0002
	CD56+^{dim}TLR-3+[%]					
	I vs. II	I vs. III	I vs. IV	II vs. III	II vs. IV	III vs. IV
**Area**	1	1	1	0.9685	1	0.9375
**Std. Error**	0	0	0	0.02313	0	0.02943
**95% confidence interval**	1,000 to 1,000	1,000 to 1,000	1,000 to 1,000	0,9232 to 1,000	1,000 to 1,000	0,8798 to 0,9952
**P value**	<0,0001	<0,0001	<0,0001	<0,0001	<0,0001	<0,0001
	CD56+^{dim}TLR-4+[%]					
	I vs. II	I vs. III	I vs. IV	II vs. III	II vs. IV	III vs. IV
**Area**	0.9156	1	1	0.9944	1	0.6354
**Std. Error**	0.05302	0	0	0.006859	0	0.07428
**95% confidence interval**	0,8116 to 1,000	1,000 to 1,000	1,000 to 1,000	0,9810 to 1,000	1,000 to 1,000	0,4898 to 0,7810
**P value**	0.0001	<0,0001	<0,0001	<0,0001	<0,0001	0.0955
	CD56+^{dim}TLR-9+ [%]					
	I vs. II	I vs. III	I vs. IV	II vs. III	II vs. IV	III vs. IV
**Area**	0.8267	1	1	0.962	1	0.7819
**Std. Error**	0.07459	0	0	0.02461	0	0.06291
**95% confidence interval**	0,6805 to 0,9729	1,000 to 1,000	1,000 to 1,000	0,9138 to 1,000	1,000 to 1,000	0,6587 to 0,9052
**P value**	0.0023	<0,0001	<0,0001	<0,0001	<0,0001	0.0005
	CD56+^{bright}TLR-2+[%]					
	I vs. II	I vs. III	I vs. IV	II vs. III	II vs. IV	III vs. IV
**Area**	0.84	1	1	0.9667	1	0.7653
**Std. Error**	0.07913	0	0	0.02115	0	0.06298
**95% confidence interval**	0,6849 to 0,9951	1,000 to 1,000	1,000 to 1,000	0,9252 to 1,000	1,000 to 1,000	0,6418 to 0,8887
**P value**	0.0015	<0,0001	<0,0001	<0,0001	<0,0001	0.0011
	CD56+^{bright}TLR-3+[%]					
	I vs. II	I vs. III	I vs. IV	II vs. III	II vs. IV	III vs. IV

**Area**	0.9956	1	1	0.8519	1	0.8403
**Std. Error**	0.007354	0	0	0.05294	0	0.05305
**95% confidence interval**	0,9811 to 1,000	1,000 to 1,000	1,000 to 1,000	0,7481 to 0,9556	1,000 to 1,000	0,7363 to 0,9443
**P value**	<0,0001	<0,0001	<0,0001	<0,0001	<0,0001	<0,0001
	CD56+brightTLR-4+[%]					
	I vs. II	I vs. III	I vs. IV	II vs. III	II vs. IV	III vs. IV
**Area**	0.9378	1	1	0.9185	1	0.6028
**Std. Error**	0.04543	0	0	0.03795	0	0.07761
**95% confidence interval**	0,8487 to 1,000	1,000 to 1,000	1,000 to 1,000	0,8441 to 0,9929	1,000 to 1,000	0,4507 to 0,7549
**P value**	<0,0001	<0,0001	<0,0001	<0,0001	<0,0001	0.2057
	CD56+brightTLR-9+[%]					
	I vs. II	I vs. III	I vs. IV	II vs. III	II vs. IV	III vs. IV
**Area**	0.8756	1	1	0.9444	0.99	0.7569
**Std. Error**	0.06218	0	0	0.03158	0.01125	0.06713
**95% confidence interval**	0,7537 to 0,9974	1,000 to 1,000	1,000 to 1,000	0,8826 to 1,000	0,9679 to 1,000	0,6254 to 0,8885
**P value**	0.0005	<0,0001	<0,0001	<0,0001	<0,0001	0.0016
	CD3+CD56+TLR-2+[%]					
	I vs. II	I vs. III	I vs. IV	II vs. III	II vs. IV	III vs. IV
**Area**	0.92	1	1	1	1	0.9
**Std. Error**	0.0498	0	0	0	0	0.04902
**95% confidence interval**	0,8224 to 1,000	1,000 to 1,000	1,000 to 1,000	1,000 to 1,000	1,000 to 1,000	0,8039 to 0,9961
**P value**	<0,0001	<0,0001	<0,0001	<0,0001	<0,0001	<0,0001
	CD3+CD56+TLR-3+[%]					
	I vs. II	I vs. III	I vs. IV	II vs. III	II vs. IV	III vs. IV
**Area**	0.96	1	1	0.6259	0.93	0.9139
**Std. Error**	0.0311	0	0	0.1035	0.04126	0.04541
**95% confidence interval**	0,8990 to 1,000	1,000 to 1,000	1,000 to 1,000	0,4230 to 0,8288	0,8491 to 1,000	0,8249 to 1,000
**P value**	<0,0001	<0,0001	<0,0001	0.1598	<0,0001	<0,0001
	CD3+CD56+TLR-4+[%]					
	I vs. II	I vs. III	I vs. IV	II vs. III	II vs. IV	III vs. IV
**Area**	0.6778	1	1	0.9907	1	0.8889
**Std. Error**	0.1036	0	0	0.01042	0	0.04615
**95% confidence interval**	0,4747 to 0,8809	1,000 to 1,000	1,000 to 1,000	0,9703 to 1,000	1,000 to 1,000	0,7984 to 0,9793
**P value**	0.0971	<0,0001	<0,0001	<0,0001	<0,0001	<0,0001
	CD3+CD56+TLR-9+[%]					
	I vs. II	I vs. III	I vs. IV	II vs. III	II vs. IV	III vs. IV

**Area**	1	1	1	0.9241	1	0.925
**Std. Error**	0	0	0	0.04241	0	0.03558
**95% confidence interval**	1,000 to 1,000	1,000 to 1,000	1,000 to 1,000	0,8410 to 1,000	1,000 to 1,000	0,8553 to 0,9947
**P value**	<0,0001	<0,0001	<0,0001	<0,0001	<0,0001	<0,0001
	TLR-2 expression in NK cells					
	I vs. II	I vs. III	I vs. IV	II vs. III	II vs. IV	III vs. IV
**Area**	0.9111	1	1	0.9704	1	0.9069
**Std. Error**	0.06105	0	0	0.01943	0	0.04038
**95% confidence interval**	0,7914 to 1,000	1,000 to 1,000	1,000 to 1,000	0,9323 to 1,000	1,000 to 1,000	0,8278 to 0,9861
**P value**	0.0001	<0,0001	<0,0001	<0,0001	<0,0001	<0,0001
	TLR-3 expression in NK cells					
	I vs. II	I vs. III	I vs. IV	II vs. III	II vs. IV	III vs. IV
**Area**	0.8222	0.9778	1	0.8556	1	0.8833
**Std. Error**	0.08055	0.01688	0	0.05216	0	0.04566
**95% confidence interval**	0,6644 to 0,9801	0,9447 to 1,000	1,000 to 1,000	0,7533 to 0,9578	1,000 to 1,000	0,7938 to 0,9728
**P value**	0.0026	<0,0001	<0,0001	<0,0001	<0,0001	<0,0001
	TLR-4 expression in NK cells					
	I vs. II	I vs. III	I vs. IV	II vs. III	II vs. IV	III vs. IV
**Area**	0.8356	1	1	0.987	1	0.6194
**Std. Error**	0.0776	0	0	0.01148	0	0.07528
**95% confidence interval**	0,6835 to 0,9876	1,000 to 1,000	1,000 to 1,000	0,9645 to 1,000	1,000 to 1,000	0,4719 to 0,7670
**P value**	0.0017	<0,0001	<0,0001	<0,0001	<0,0001	0.1414
	TLR-9 expression in NK cells					
	I vs. II	I vs. III	I vs. IV	II vs. III	II vs. IV	III vs. IV
**Area**	0.7511	0.9926	1	0.963	1	0.8736
**Std. Error**	0.09037	0.007805	0	0.02267	0	0.04986
**95% confidence interval**	0,5740 to 0,9282	0,9773 to 1,000	1,000 to 1,000	0,9185 to 1,000	1,000 to 1,000	0,7759 to 0,9713
**P value**	0.0191	<0,0001	<0,0001	<0,0001	<0,0001	<0,0001
	TLR-2 expression in NKT-like cells					
	I vs. II	I vs. III	I vs. IV	II vs. III	II vs. IV	III vs. IV
**Area**	0.5711	0.9722	1	0.9463	1	0.9139
**Std. Error**	0.1079	0.01859	0	0.03524	0	0.03745
**95% confidence interval**	0,3597 to 0,7825	0,9358 to 1,000	1,000 to 1,000	0,8772 to 1,000	1,000 to 1,000	0,8405 to 0,9873
**P value**	0.5069	<0,0001	<0,0001	<0,0001	<0,0001	<0,0001
	TLR-3 expression in NKT-like cells					
	I vs. II	I vs. III	I vs. IV	II vs. III	II vs. IV	III vs. IV

**Area**	0.8667	0.9204	0.93	0.65	0.6933	0.5306
**Std. Error**	0.06453	0.03768	0.04028	0.08071	0.09174	0.08001
**95% confidence interval**	0,7402 to 0,9931	0,8465 to 0,9942	0,8511 to 1,000	0,4918 to 0,8082	0,5135 to 0,8731	0,3737 to 0,6874
**P value**	0.0006	<0,0001	<0,0001	0.094	0.0532	0.7068
	TLR-4 expression in NKT-like cells					
	I vs. II	I vs. III	I vs. IV	II vs. III	II vs. IV	III vs. IV
**Area**	0.6667	0.8796	0.9767	0.6944	0.87	0.6653
**Std. Error**	0.1032	0.0463	0.02082	0.08286	0.05827	0.07451
**95% confidence interval**	0,4644 to 0,8689	0,7889 to 0,9704	0,9359 to 1,000	0,5320 to 0,8569	0,7558 to 0,9842	0,5192 to 0,8113
**P value**	0.1198	<0,0001	<0,0001	0.03	0.0002	0.0419
	TLR-9 expression in NKT-like cells					
	I vs. II	I vs. III	I vs. IV	II vs. III	II vs. IV	III vs. IV
**Area**	0.8844	0.987	0.9933	0.8704	0.9367	0.6882
**Std. Error**	0.0617	0.01394	0.008953	0.05287	0.0378	0.08024
**95% confidence interval**	0,7635 to 1,000	0,9597 to 1,000	0,9758 to 1,000	0,7668 to 0,9740	0,8626 to 1,000	0,5309 to 0,8455
**P value**	0.0003	<0,0001	<0,0001	<0,0001	<0,0001	0.0205

Supplementary materials Table S8. Tabulated summary of ROC curves for diffuse and intestinal type of GC and HV

	CD3-**CD56+^{dim}TLR-2+[%]**		
	D vs. I	D vs. HV	I vs. HV
**Area**	0.6644	1	1
**Std. Error**	0.05868	0	0
**95% confidence interval**	0,5494 to 0,7794	1,000 to 1,000	1,000 to 1,000
**P value**	0.0099	<0,0001	<0,0001

	**CD3-**CD56+^{dim}TLR-3+[%]		
	D vs. I	D vs. HV	I vs. HV
**Area**	0.5938	1	1
**Std. Error**	0.06284	0	0
**95% confidence interval**	0,4707 to 0,7170	1,000 to 1,000	1,000 to 1,000
**P value**	0.1409	<0,0001	<0,0001

	**CD3-**CD56+^{dim}TLR-4+[%]		
	D vs. I	D vs. HV	I vs. HV
**Area**	0.5782	1	1
**Std. Error**	0.06383	0	0
**95% confidence interval**	0,4530 to 0,7033	1,000 to 1,000	1,000 to 1,000
**P value**	0.2201	<0,0001	<0,0001

	**CD3-**CD56+^{dim}TLR-9+[%]		
	D vs. I	D vs. HV	I vs. HV
**Area**	0.6073	1	1
**Std. Error**	0.06306	0	0
**95% confidence interval**	0,4837 to 0,7309	1,000 to 1,000	1,000 to 1,000
**P value**	0.0923	<0,0001	<0,0001

	CD56+^{bright}TLR-2+[%]		
	D vs. I	D vs. HV	I vs. HV
**Area**	0.6577	1	1
**Std. Error**	0.06009	0	0
**95% confidence interval**	0,5399 to 0,7755	1,000 to 1,000	1,000 to 1,000
**P value**	0.0133	<0,0001	<0,0001

	**CD3-**CD56+^{bright}TLR-3+[%]		
	D vs. I	D vs. HV	I vs. HV
**Area**	0.5938	1	1
**Std. Error**	0.06261	0	0

**95% confidence interval**	0,4711 to 0,7166	1,000 to 1,000	1,000 to 1,000
**P value**	0.1409	<0,0001	<0,0001

	**CD3-**CD56+^{bright}TLR-4+[%]		
	D vs. I	D vs. HV	I vs. HV
**Area**	0.5927	1	1
**Std. Error**	0.06297	0	0
**95% confidence interval**	0,4693 to 0,7161	1,000 to 1,000	1,000 to 1,000
**P value**	0.1457	<0,0001	<0,0001

	**CD3-**CD56+^{bright}TLR-9+[%]		
	D vs. I	D vs. HV	I vs. HV
**Area**	0.6202	1	1
**Std. Error**	0.06223	0	0
**95% confidence interval**	0,4982 to 0,7421	1,000 to 1,000	1,000 to 1,000
**P value**	0.0594	<0,0001	<0,0001

	CD3+CD56+TLR-2+ [%]		
	D vs. I	D vs. HV	I vs. HV
**Area**	0.6025	1	1
**Std. Error**	0.06156	0	0
**95% confidence interval**	0,4819 to 0,7232	1,000 to 1,000	1,000 to 1,000
**P value**	0.1077	<0,0001	<0,0001

	CD3+CD56+TLR-3+ [%]		
	D vs. I	D vs. HV	I vs. HV
**Area**	0.5647	1	1
**Std. Error**	0.06369	0	0
**95% confidence interval**	0,4399 to 0,6895	1,000 to 1,000	1,000 to 1,000
**P value**	0.31	<0,0001	<0,0001

	CD3+CD56+TLR-4+ [%]		
	D vs. I	D vs. HV	I vs. HV
**Area**	0.6025	1	1
**Std. Error**	0.0622	0	0
**95% confidence interval**	0,4806 to 0,7244	1,000 to 1,000	1,000 to 1,000
**P value**	0.1077	<0,0001	<0,0001

	CD3+CD56+TLR-9+ [%]		
	D vs. I	D vs. HV	I vs. HV
**Area**	0.6375	1	1
**Std. Error**	0.05955	0	0
**95% confidence interval**	0,5208 to 0,7542	1,000 to 1,000	1,000 to 1,000
**P value**	0.0309	<0,0001	<0,0001

	TLR-2 expression in NK cells		
	D vs. I	D vs. HV	I vs. HV
**Area**	0.7132	1	1
**Std. Error**	0.05473	0	0
**95% confidence interval**	0,6059 to 0,8204	1,000 to 1,000	1,000 to 1,000
**P value**	0.0008	<0,0001	<0,0001

	TLR-3 expression in NK cells		
	D vs. I	D vs. HV	I vs. HV
**Area**	0.7059	1	1
**Std. Error**	0.0555	0	0
**95% confidence interval**	0,5971 to 0,8147	1,000 to 1,000	1,000 to 1,000
**P value**	0.0012	<0,0001	<0,0001

	TLR-4 expression in NK cells		
	D vs. I	D vs. HV	I vs. HV
**Area**	0.7305	1	1
**Std. Error**	0.05323	0	0
**95% confidence interval**	0,6262 to 0,8349	1,000 to 1,000	1,000 to 1,000
**P value**	0.0003	<0,0001	<0,0001

	TLR-9 expression in NK cells		
	D vs. I	D vs. HV	I vs. HV
**Area**	0.702	1	1
**Std. Error**	0.05587	0	0
**95% confidence interval**	0,5925 to 0,8115	1,000 to 1,000	1,000 to 1,000
**P value**	0.0015	<0,0001	<0,0001

	TLR-2 expression in NKT-like cells		
	D vs. I	D vs. HV	I vs. HV
**Area**	0.637	1	1
**Std. Error**	0.05994	0	0
**95% confidence interval**	0,5195 to 0,7545	1,000 to 1,000	1,000 to 1,000
**P value**	0.0316	<0,0001	<0,0001

	TLR-3 expression in NKT-like cells		
	D vs. I	D vs. HV	I vs. HV
**Area**	0.516	1	1
**Std. Error**	0.06374	0	0
**95% confidence interval**	0,3910 to 0,6409	1,000 to 1,000	1,000 to 1,000
**P value**	0.8022	<0,0001	<0,0001

	TLR-4 expression in NKT-like cells		
	D vs. I	D vs. HV	I vs. HV
**Area**	0.5389	1	1

**Std. Error**	0.0634	0	0
**95% confidence interval**	0,4147 to 0,6632	1,000 to 1,000	1,000 to 1,000
**P value**	0.5412	<0,0001	<0,0001

	TLR-9 expression in NKT-like cells		
	D vs. I	D vs. HV	I vs. HV
**Area**	0.6168	1	1
**Std. Error**	0.0608	0	0
**95% confidence interval**	0,4977 to 0,7360	1,000 to 1,000	1,000 to 1,000
**P value**	0.0668	<0,0001	<0,0001

Supplementary materials Tabel S9. Tabulated summary of ROC curves for age categories of GC and HV patients

	CD3-**CD56+^{dim}TLR-2+ [%]**					
	I vs. II	I vs. III	I vs. IV	II vs. III	II vs. IV	III vs. IV
**Area**	0.5663	1	1	1	1	0.7245
**Std. Error**	0.06534	0	0	0	0	0.09842
**95% confidence interval**	0,4383 to 0,6944	1,000 to 1,000	1,000 to 1,000	1,000 to 1,000	1,000 to 1,000	0,5316 to 0,9174
**P value**	0.3029	<0,0001	<0,0001	<0,0001	<0,0001	0.0401
	**CD3-**CD56+^{dim}TLR-3+ [%]					
	I vs. II	I vs. III	I vs. IV	II vs. III	II vs. IV	III vs. IV
**Area**	0.5615	1	1	1	1	0.6273
**Std. Error**	0.06284	0	0	0	0	0.1074
**95% confidence interval**	0,4383 to 0,6846	1,000 to 1,000	1,000 to 1,000	1,000 to 1,000	1,000 to 1,000	0,4169 to 0,8378
**P value**	0.3398	<0,0001	<0,0001	<0,0001	<0,0001	0.2444
	**CD3-**CD56+^{dim}TLR-4+ [%]					
	I vs. II	I vs. III	I vs. IV	II vs. III	II vs. IV	III vs. IV
**Area**	0.6006	1	1	1	1	0.6273
**Std. Error**	0.06173	0	0	0	0	0.119
**95% confidence interval**	0,4796 to 0,7216	1,000 to 1,000	1,000 to 1,000	1,000 to 1,000	1,000 to 1,000	0,3942 to 0,8605
**P value**	0.1181	<0,0001	<0,0001	<0,0001	<0,0001	0.2444
	**CD3-**CD56+^{dim}TLR-9+ [%]					
	I vs. II	I vs. III	I vs. IV	II vs. III	II vs. IV	III vs. IV
**Area**	0.6338	1	1	1	1	0.6481
**Std. Error**	0.06245	0	0	0	0	0.1003
**95% confidence interval**	0,5114 to 0,7562	1,000 to 1,000	1,000 to 1,000	1,000 to 1,000	1,000 to 1,000	0,4517 to 0,8446
**P value**	0.0377	<0,0001	<0,0001	<0,0001	<0,0001	0.1755
	**CD3-**CD56+^{bright}TLR-2+ [%]					
	I vs. II	I vs. III	I vs. IV	II vs. III	II vs. IV	III vs. IV
**Area**	0.5895	1	1	1	1	0.5532
**Std. Error**	0.06538	0	0	0	0	0.1097
**95% confidence interval**	0,4613 to 0,7176	1,000 to 1,000	1,000 to 1,000	1,000 to 1,000	1,000 to 1,000	0,3382 to 0,7683
**P value**	0.1646	<0,0001	<0,0001	<0,0001	<0,0001	0.6264
	**CD3-**CD56+^{bright}TLR-3+ [%]					
	I vs. II	I vs. III	I vs. IV	II vs. III	II vs. IV	III vs. IV
**Area**	0.5655	1	1	1	1	0.6181
**Std. Error**	0.06308	0	0	0	0	0.1127

**95% confidence interval**	0,4418 to 0,6891	1,000 to 1,000	1,000 to 1,000	1,000 to 1,000	1,000 to 1,000	0,3971 to 0,8390
**P value**	0.3092	<0,0001	<0,0001	<0,0001	<0,0001	0.2804
	**CD3-**CD56+^{bright}TLR-4+ [%]					
	I vs. II	I vs. III	I vs. IV	II vs. III	II vs. IV	III vs. IV
**Area**	0.6006	1	1	1	1	0.6088
**Std. Error**	0.06217	0	0	0	0	0.1202
**95% confidence interval**	0,4788 to 0,7225	1,000 to 1,000	1,000 to 1,000	1,000 to 1,000	1,000 to 1,000	0,3732 to 0,8444
**P value**	0.1181	<0,0001	<0,0001	<0,0001	<0,0001	0.3198
	**CD3-**CD56+^{bright}TLR-9+ [%]					
	I vs. II	I vs. III	I vs. IV	II vs. III	II vs. IV	III vs. IV
**Area**	0.6404	1	1	1	1	0.6713
**Std. Error**	0.06212	0	0	0	0	0.0997
**95% confidence interval**	0,5186 to 0,7621	1,000 to 1,000	1,000 to 1,000	1,000 to 1,000	1,000 to 1,000	0,4759 to 0,8667
**P value**	0.0292	<0,0001	<0,0001	<0,0001	<0,0001	0.1173
	CD3+CD56+TLR-2+ [%]					
	I vs. II	I vs. III	I vs. IV	II vs. III	II vs. IV	III vs. IV
**Area**	0.5989	1	1	1	1	0.6481
**Std. Error**	0.06189	0	0	0	0	0.1003
**95% confidence interval**	0,4776 to 0,7202	1,000 to 1,000	1,000 to 1,000	1,000 to 1,000	1,000 to 1,000	0,4517 to 0,8446
**P value**	0.1245	<0,0001	<0,0001	<0,0001	<0,0001	0.1755
	CD3+CD56+TLR-3+ [%]					
	I vs. II	I vs. III	I vs. IV	II vs. III	II vs. IV	III vs. IV
**Area**	0.5678	1	1	1	1	0.6481
**Std. Error**	0.06373	0	0	0	0	0.1052
**95% confidence interval**	0,4428 to 0,6927	1,000 to 1,000	1,000 to 1,000	1,000 to 1,000	1,000 to 1,000	0,4420 to 0,8543
**P value**	0.2926	<0,0001	<0,0001	<0,0001	<0,0001	0.1755
	CD3+CD56+TLR-4+ [%]					
	I vs. II	I vs. III	I vs. IV	II vs. III	II vs. IV	III vs. IV
**Area**	0.5981	1	1	1	1	0.5856
**Std. Error**	0.06341	0	0	0	0	0.1093
**95% confidence interval**	0,4738 to 0,7223	1,000 to 1,000	1,000 to 1,000	1,000 to 1,000	1,000 to 1,000	0,3714 to 0,7998
**P value**	0.1278	<0,0001	<0,0001	<0,0001	<0,0001	0.4335
	CD3+CD56+TLR-9+ [%]					
	I vs. II	I vs. III	I vs. IV	II vs. III	II vs. IV	III vs. IV
**Area**	0.5374	1	1	1	1	0.6412
**Std. Error**	0.06459	0	0	0	0	0.105

**95% confidence interval**	0,4109 to 0,6640	1,000 to 1,000	1,000 to 1,000	1,000 to 1,000	1,000 to 1,000	0,4354 to 0,8471
**P value**	0.5608	<0,0001	<0,0001	<0,0001	<0,0001	0.1966
	TLR-2 expression in NK cells					
	I vs. II	I vs. III	I vs. IV	II vs. III	II vs. IV	III vs. IV
**Area**	0.5663	1	1	1	1	0.537
**Std. Error**	0.06441	0	0	0	0	0.1074
**95% confidence interval**	0,4401 to 0,6926	1,000 to 1,000	1,000 to 1,000	1,000 to 1,000	1,000 to 1,000	0,3265 to 0,7476
**P value**	0.3029	<0,0001	<0,0001	<0,0001	<0,0001	0.7349
	TLR-3 expression in NK cells					
	I vs. II	I vs. III	I vs. IV	II vs. III	II vs. IV	III vs. IV
**Area**	0.5569	1	1	1	1	0.537
**Std. Error**	0.06385	0	0	0	0	0.1072
**95% confidence interval**	0,4318 to 0,6820	1,000 to 1,000	1,000 to 1,000	1,000 to 1,000	1,000 to 1,000	0,3269 to 0,7471
**P value**	0.3769	<0,0001	<0,0001	<0,0001	<0,0001	0.7349
	TLR-4 expression in NK cells					
	I vs. II	I vs. III	I vs. IV	II vs. III	II vs. IV	III vs. IV
**Area**	0.5695	1	1	1	1	0.5324
**Std. Error**	0.06383	0	0	0	0	0.108
**95% confidence interval**	0,4444 to 0,6946	1,000 to 1,000	1,000 to 1,000	1,000 to 1,000	1,000 to 1,000	0,3207 to 0,7441
**P value**	0.2806	<0,0001	<0,0001	<0,0001	<0,0001	0.767
	TLR-9 expression in NK cells					
	I vs. II	I vs. III	I vs. IV	II vs. III	II vs. IV	III vs. IV
**Area**	0.5592	1	1	1	1	0.537
**Std. Error**	0.06403	0	0	0	0	0.1072
**95% confidence interval**	0,4337 to 0,6847	1,000 to 1,000	1,000 to 1,000	1,000 to 1,000	1,000 to 1,000	0,3269 to 0,7471
**P value**	0.358	<0,0001	<0,0001	<0,0001	<0,0001	0.7349
	TLR-2 expression in NKT-like cells					
	I vs. II	I vs. III	I vs. IV	II vs. III	II vs. IV	III vs. IV
**Area**	0.586	1	1	1	1	0.7037
**Std. Error**	0.06301	0	0	0	0	0.1024
**95% confidence interval**	0,4626 to 0,7095	1,000 to 1,000	1,000 to 1,000	1,000 to 1,000	1,000 to 1,000	0,5031 to 0,9044
**P value**	0.1814	<0,0001	<0,0001	<0,0001	<0,0001	0.0625
	TLR-3 expression in NKT-like cells					
	I vs. II	I vs. III	I vs. IV	II vs. III	II vs. IV	III vs. IV
**Area**	0.5143	1	1	1	1	0.5023
**Std. Error**	0.06425	0	0	0	0	0.1182

**95% confidence interval**	0,3884 to 0,6402	1,000 to 1,000	1,000 to 1,000	1,000 to 1,000	1,000 to 1,000	0,2707 to 0,7339
**P value**	0.8243	<0,0001	<0,0001	<0,0001	<0,0001	0.9831
	TLR-4 expression in NKT-like cells					
	I vs. II	I vs. III	I vs. IV	II vs. III	II vs. IV	III vs. IV
**Area**	0.518	1	1	1	1	0.537
**Std. Error**	0.06359	0	0	0	0	0.1141
**95% confidence interval**	0,3934 to 0,6426	1,000 to 1,000	1,000 to 1,000	1,000 to 1,000	1,000 to 1,000	0,3134 to 0,7607
**P value**	0.7797	<0,0001	<0,0001	<0,0001	<0,0001	0.7349
	TLR-9 expression in NKT-like cells					
	I vs. II	I vs. III	I vs. IV	II vs. III	II vs. IV	III vs. IV
**Area**	0.562	1	1	1	1	0.5625
**Std. Error**	0.06359	0	0	0	0	0.1069
**95% confidence interval**	0,4374 to 0,6867	1,000 to 1,000	1,000 to 1,000	1,000 to 1,000	1,000 to 1,000	0,3531 to 0,7719
**P value**	0.3353	<0,0001	<0,0001	<0,0001	<0,0001	0.5677

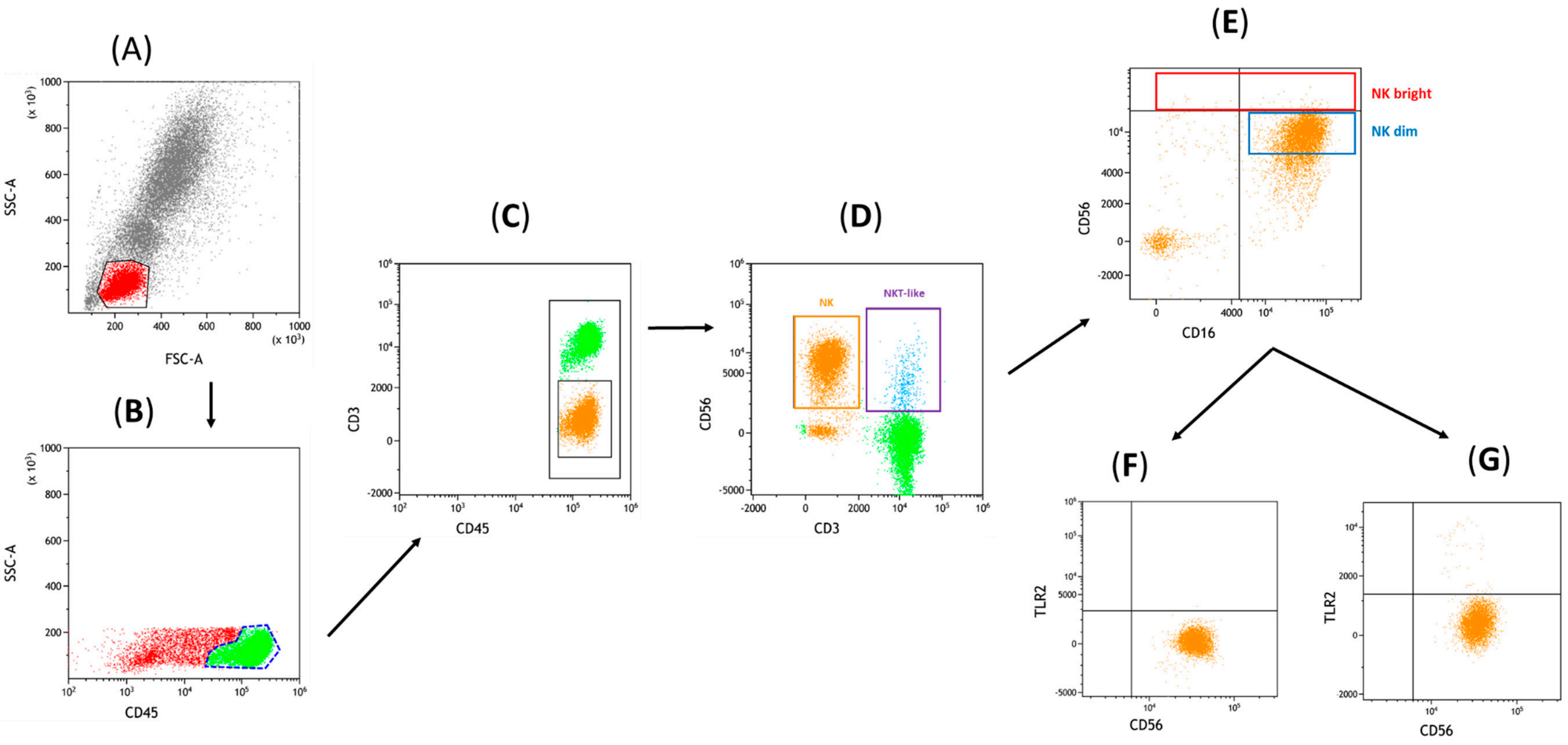

Supplementary Figure S1. Example of flow cytometric analysis. In the first step (A), lymphocytes were selected based on the parameters FSC-A (Forward Scatter Area) and SSC-A (Side Scatter Area), which allowed for the identification of the appropriate cells. Then, in step (B), gating for CD45+ cells was performed, which allowed for the isolation of leukocytes, i.e. cells characterized by the expression of this marker. The next step (C) was the selection of cells expressing both CD3 and CD45, which allowed for the distinction of T cells from other leukocytes. In step (D), the expression of CD3 and CD56 markers was analyzed, which allowed for the identification of two main populations: NK cells (natural cytotoxic cells) and NKT-like cells. NK cells (orange) express CD56 but not CD3, whereas NKT-like cells (blue) express both CD3 and CD56. In the following analysis, steps (E) focus on the detailed characterization of NK cells, distinguishing between bright NK cells (high CD56 expression) and dim NK cells (low CD56 expression), while also taking into account CD16 expression. In the following

analysis, steps (F) Represent FMO control for gating CD3-CD56+ cells expressing the TLR2 receptor within the NK^{dim} population. (G) Represents an example analysis for CD3-CD56+ cells within the NK^{dim} population expressing the TLR2 receptor.

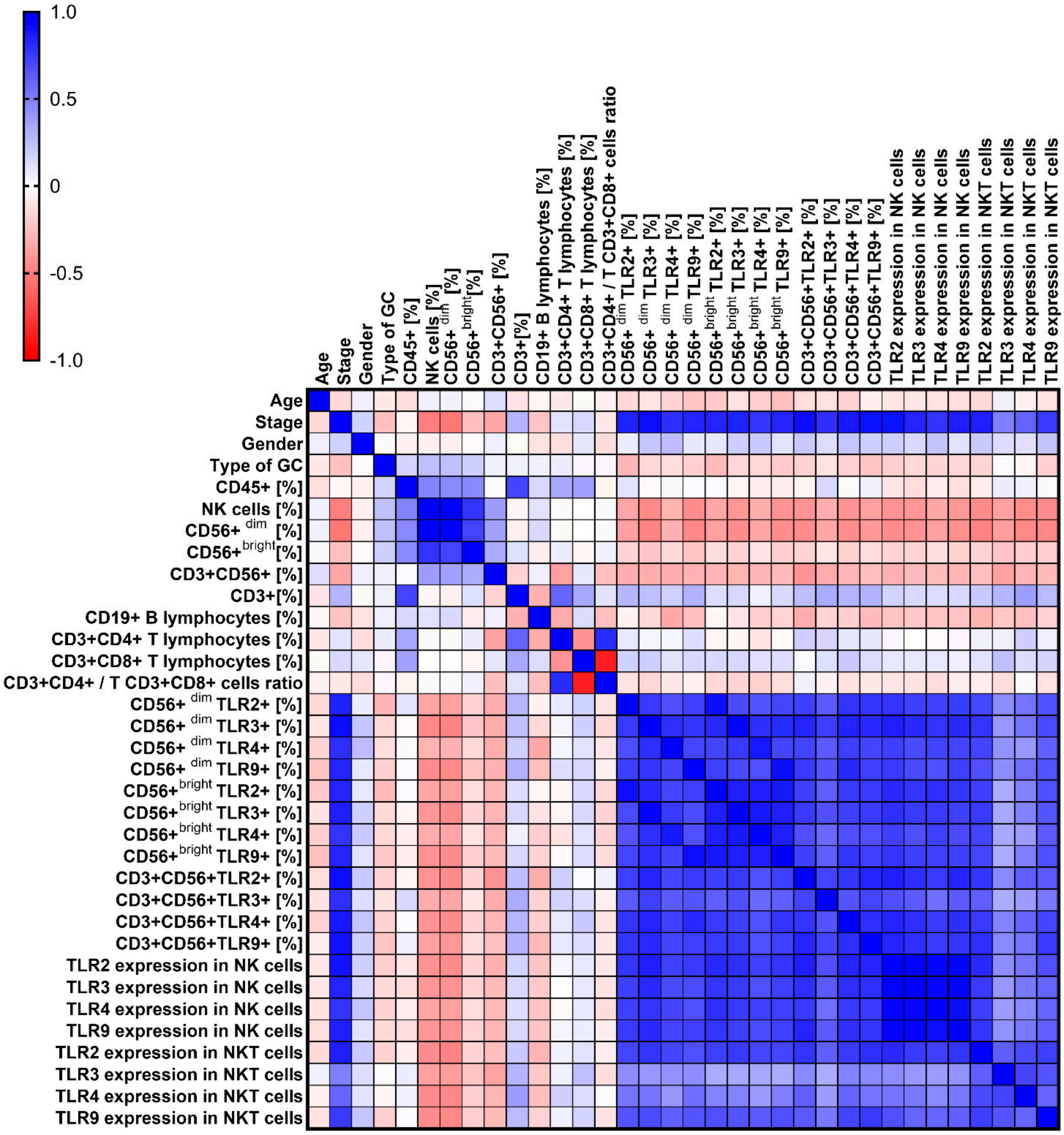

Supplementary Figure S2. Schematic representation of Spearman rank correlations for individual clinical and immunological parameters of newly diagnosed GC patients.
